# Bifunctional Luminomagnetic Rare-Earth Nanorods for High-Contrast Bioimaging Nanoprobes

**DOI:** 10.1038/srep32401

**Published:** 2016-09-02

**Authors:** Bipin Kumar Gupta, Satbir Singh, Pawan Kumar, Yean Lee, Garima Kedawat, Tharangattu N. Narayanan, Sajna Antony Vithayathil, Liehui Ge, Xiaobo Zhan, Sarika Gupta, Angel A. Martí, Robert Vajtai, Pulickel M. Ajayan, Benny Abraham Kaipparettu

**Affiliations:** 1 Luminescent Materials and Devices Group, Materials Physics and Engineering Division, CSIR- National Physical Laboratory, Dr K S Krishnan Road, New Delhi, 110012, India; 2 Academy of Scientific and Innovative Research (AcSIR), CSIR-National Physical Laboratory Campus, Dr K S Krishnan Road, New Delhi 110012, India; 3 Department of Material Science and Nano Engineering Rice University, Houston, TX 77005, USA; 4 Department of Physics, Kalindi College, University of Delhi, New Delhi, 110008, India; 5 TIFR- Center for Interdisciplinary sciences, Tata Institute fundamental research, Hydrabad-500075, India; 6 Department of Molecular and Human Genetics, Baylor College of Medicine, Houston, TX 77030, USA; 7 National Institute of Immunology, Aruna Aseaf Ali Marg, J. N. U. Complex, New Delhi-110067, India; 8 Department of Chemistry and Bioengineering, Rice University, Houston, Texas 77005, USA; 9 Dan L. Duncan Cancer Center, Baylor College of Medicine, Houston, TX 77030, USA

## Abstract

Nanoparticles exhibiting both magnetic and luminescent properties are need of the hour for many biological applications. A single compound exhibiting this combination of properties is uncommon. Herein, we report a strategy to synthesize a bifunctional luminomagnetic Gd_2−x_Eu_x_O_3_ (x = 0.05 to 0.5) nanorod, with a diameter of ~20 nm and length in ~0.6 μm, using hydrothermal method. Gd_2_O_3_:Eu^3+^ nanorods have been characterized by studying its structural, optical and magnetic properties. The advantage offered by photoluminescent imaging with Gd_2_O_3_:Eu^3+^ nanorods is that this ultrafine nanorod material exhibits hypersensitive intense red emission (610 nm) with good brightness (quantum yield more than 90%), which is an essential parameter for high-contrast bioimaging, especially for overcoming auto fluorescent background. The utility of luminomagnetic nanorods for biological applications in high-contrast cell imaging capability and cell toxicity to image two human breast cancer cell lines T47D and MDA-MB-231 are also evaluated. Additionally, to understand the significance of shape of the nanostructure, the photoluminescence and paramagnetic characteristic of Gd_2_O_3_:Eu^3+^ nanorods were compared with the spherical nanoparticles of Gd_2_O_3_:Eu^3+^.

Bifunctional nanomaterials that possess desirable properties in a single entity have been the focus of cutting edge science in recent years^1^,^2^,^3^,^4^,^5^. For instance, nanomaterials with bifunctional properties such as luminescence, magnetism and high contrast bioimaging with magnetic resonance imaging (MRI) contrast capability can be used in a wide range of applications in biological systems such as bioimaging, diagnostic, and therapeutics^2^,^3^,^4^,^6^,^7^,^8^. Such nanomaterials can serve as luminescent nanoprobes that can be controlled by an external magnetic field^4^. Recent studies have shown the availability of highly efficient engineered nanostructures to task for close biological barriers, which have triggered the applications in drug delivery and controlled release as active surface in plants and as biomarkers in cell tracking or diagnostic studies. Luminescent inorganic materials posses high photostability whereas standard fluorescent organic dyes based bioimaging suffer from color fading resulting in temporal and limited use^4^. The growing potential and interest in the application of engineered nanostructures for drug delivery and other biomedical applications (for example, coatings on plants, biomarkers and diagnostic) have enhanced the likelihood of nanobio-interaction which are difficult to predict due to lack of systematic and diverse range of engineered nanomaterials that have become a challenge in the past decade^8^. Semiconductor nanoparticles having strong luminescent properties, for example, CdSe have become promising alternatives to the use of organic dyes. However, their inherent toxicity limits their applications. Alternative approaches to fabricate structures such as CdSe/ZnS or CdSe and CdTe, on luminescent quantum dots which are biocompatible with cell have been only marginally successful so far^6^,^7^. Furthermore, the on and off switch of their luminescence, also known as flickering and blinking, is detrimental for their potential applications^3^,^9^. In this context, lanthanide-based inorganic materials with outstanding optical and magnetic properties are interesting alternatives for applications such as biomarkers, sensors, and contrast agents in MRI. Rare earth-doped inorganic nanoparticles maintain a photoluminescence (PL) characterized by high photochemical stability, sharp emission bands, long luminescence lifetime, lower photobleaching potential, low toxicity, and high chemical stability, suitable for biolabeling applications^3^,^4^,^10^,^11^. With the exception of La^3+^ and Lu^3+^, all trivalent lanthanide ions possess unpaired electrons resulting in paramagnetic behavior, among which gadolinium containing compounds are preferred as contrasting agents in medical diagnostics due to the high magnetic moment of Gd^4^,^12^,^13^,^14^,^15^. Recently for bio-applications including investigations, metal oxide nanoparticles such as TiO_2_, ZnO, Fe_3_O_4_, carbon based structures such as carbon black and carbon nanotubes, graphene quantum dots as well as plasmonic metal nanoparticle (gold, silver or aluminum) have been studied^2^,^8^,^16^,^17^,^18^,^19^,^20^. Among rare earth containing compounds, gadolinium based MRI contrast agents have been studied extensively but so far only very few have been reported about lanthanide oxides^21^,^22^,^23^,^24^. Besides size, morphology also plays an important role in bioimaging application of nanomaterials. For instance, one dimensional structure such as nanowires and nanorods with several hundred nanometers in length and few tenths of nanometer in diameter may interact with cells in a different manner than isotropic nanoparticles^15^,^25^. While particles may cross the cell membrane if their size is small enough, nanorods and nanowires fulfill this requirement at least in one dimension (length too large to be completely taken by the cell). The different size and shapes of the nanoparticles certainly have an influence and important place in various biomedical applications. While nanoparticle shape has been shown to impact cellular uptake, the latest study shows that specific tissues can be targeted by controlling the shape of nanoparticles. Nanoparticles having the same material, volume, and the targeting antibody, a simple change in the shape of the nanoparticle enhances its ability to target specific tissues. It has been reported that nanorods with a high aspect ratio attach more effectively to targeted cells compared with spherical nanoparticles^26^. Superior specific attachment exhibited by rod-shaped particles offers several advantages in the field of drug delivery, particularly in the delivery of drugs such as chemotherapeutics, which are highly toxic and demand the use of targeted approaches. Here, a bifunctional rod shaped nanophosphor such as europium doped gadolinium oxide with both luminescent and magnetic properties are more suitable for bioimaging applications with external magnetic tracking capability. Gd_2_O_3_:Eu^3+^ is a highly efficient red light-emitting material^27^. Besides, Gd^3+^ ion is known to be the best metal ion in the periodic table to be used as an MRI contrast agent^4^,^12^.

The present study is aimed at the synthesis of high quality ultra-fine europium doped gadolinium oxide (Gd_2−x_Eu_x_O_3,_ x = 0.15) nanorods, with a diameter of ~20 nm and length in ~0.6 μm, using a facile base catalyzed hydrothermal process. Probing this highly luminescent-paramagnetic nanophosphor using photoluminescence, time-resolved spectroscopy, magnetization measurements, cytotoxicity assay and bioimaging applications reveal that this nanophosphor is a highly suitable nanoprobe for *in vitro* as well as *in vivo* high-contrast imaging and external magnetic tracking applications. To the best of our knowledge, this is one of the first studies to investigate the application of Gd_2_O_3_:Eu^3+^ nanorods with diameter ~20 nm as biological nanoprobe for *in vitro* as well as *in vivo* bioimaging and external magnetic tracking of nanorods for potential nuclear drug delivery applications.

## Results

**Figure 1:**
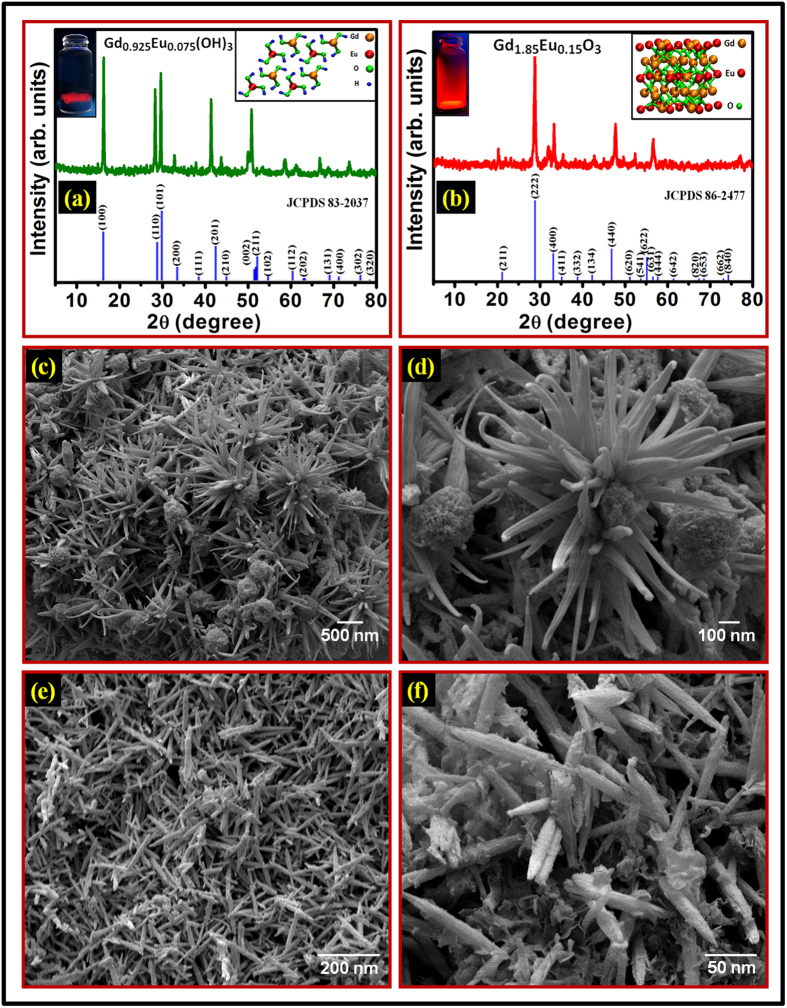
(**a**) XRD pattern of as-synthesized Gd_0.925_Eu_0.075_(OH)_3_ nanophosphor; left inset represents the as-synthesized luminescent powder sample under excitation of 254 nm and right inset exhibits the structure of Gd_0.925_Eu_0.075_(OH)_3_ nanophosphor. **(b)** XRD pattern of as-synthesized Gd_1.85_Eu_0.15_O_3_ nanorods; left inset represents the as-synthesized luminescent powder sample under excitation of 254 nm and right inset exhibits the structure of Gd_1.85_Eu_0.15_O_3_ nanorods. **(c)** SEM image of as-synthesized Gd_0.925_Eu_0.075_(OH)_3_ nanophosphor. **(d)** Magnified view of Fig. c. **(e)** SEM image of as-synthesized Gd_1.85_Eu_0.15_O_3_ nanorods. **(f)** Magnified view of Fig. e.

Gd_2−x_Eu_x_O_3_ (x = 0.05 to 0.50) nanorods were synthesized by a facile hydrothermal method as described in the experimental section. The method involves two steps; synthesis of Gd_1−y_Eu_y_(OH)_3_ (y = 0.025 to 0.250) and calcination of Gd_1−y_Eu_y_(OH)_3_ to form Gd_2−x_Eu_x_O_3_, where x = 2y. The initial amount of raw materials were taken according to empirical formula for final compound Gd_2−x_Eu_x_O_3_. Here, hydroxide is an intermediate product and its empirical formula of (Gd_1−y_Eu_y_(OH)_3_) is assumed according to final product (Gd_2−x_Eu_x_O_3_) for all hydroxide samples. The balanced chemical reactions of entire process are shown in supplementary section. The temperature and pH value of hydrothermal reaction determines the morphology of the product samples, including nanowires, nanorods, nanotubes, or nanosheets. Nanorods can be synthesized with large aspect ratio, when the pH was adjusted to 13 and the autoclave was heated at 180 °C for 24 h. The gross structural analysis and phase compositions of the samples were determined by X-ray powder diffraction (XRD). Prior to the XRD measurement, calibration of the diffractometer was done with silicon powder (*d*_111_ = 3.1353 Å)^4^. Figure 1a shows the XRD pattern of as-synthesized Gd_0.925_Eu_0.075_(OH)_3_ nanophosphor. It is well indexed to be pure hexagonal phase (space group: P6_3_/m) with estimated lattice constant of a = b = 6.3365 ±0.0067 Å and c = 3.6388 ±0.0037 Å (Joint Committee on Powder Diffraction Standards, JCPDS No. 83-2037). No additional peaks of other phases, particularly Eu(OH)_3_, have been found, revealing the formation of a pure hexagonal phase having low content of Eu. The lattice parameters for all variants of Gd_1−y_Eu_y_(OH)_3_ were calculated from observed d-values by least squares fitting using unitcell refinement software^3^,^28^. The cell parameters of other variants of Gd_1−y_Eu_y_(OH)_3_, y = 0.025 to 0.250 nanorods are shown in table TS1(see Supplementary Information). The left and right inset of Fig. 1a show the as-synthesized luminescent powder sample under excitation of 254 nm and unit cell structure of Gd_0.925_Eu_0.075_(OH)_3_ nanophosphor. After annealing at 800 °C for 3 h, the XRD pattern of as-synthesized Gd_1.85_Eu_0.15_O_3_ nanorods is shown in Fig. 1b. The intensities and positions of the peaks are changed as compared to earlier and the diffraction peaks coincide with the standard JCPDS card no. 86-2477of Gd_2_O_3_. This material was readily indexed to a pure cubic bixbyte phase with space group *Ia*^*3*^, possessing lattice constant of a = b = c = 10.8101 ± 0.0029 Å. The cell parameters of other variants of Gd_2−x_O_3_:Eu^3+^, x = 0.05 to 0.5 nanorods are shown in table TS2 (see Supplementary Information) along with the deviation of cell volume compared to the JCPDS standard. It can be noticed that the cell parameters and cell volume are increasing as a concentration of Eu increases up to x = 0.15 and decreasing thereafter. This may be due to the fact that initially, Eu substitutes in unit cell at Gd site, as a result lattice expands significantly and hence lattice parameters increase. After optimum substitution x = 0.15, the Eu atom randomly inserted in interstitial voids which results into strong Coulombic interaction between the Eu atoms in interstitial sites and oxygen atoms available in unit cell. Consequently, the unit cell shrinks and lattice parameters decreases. Such characteristics have been earlier observed in other rare-earth oxides systems by our group as well as by other researchers^3^,^4^,^28^,^29^. Sharp and strong diffraction peaks demonstrate the high crystallinity of Gd_1.85_Eu_0.15_O_3_ nanorods synthesized by this method. It also reflects the difference in growth orientation of the crystals of Gd_0.925_Eu_0.075_(OH)_3_ vs Gd_1.85_Eu_0.15_O_3_ nanophosphor. No characteristic peaks of other products were detected, implying that the Eu^3+^ ions have been effectively doped into the Gd_2_O_3_ host lattice to form solid solution after calcination. The left and right inset of Fig. 1b also show the as-synthesized luminescent powder sample under excitation of 254 nm and structure of Gd_1.85_Eu_0.15_O_3_ nanophosphor. The right inset of the Fig. 1b shows the proposed model of cubic cell of the bixbyte structure of Gd_1.85_Eu_0.15_O_3_, where an Eu atom replaces a Gd atom.

Raman spectroscopy is a very powerful tool for characterizing materials because it is an *in situ* and non-destructive method. The Raman spectra of as-synthesized Gd_0.925_Eu_0.075_(OH)_3_ nanophosphor and Gd_1.85_Eu_0.15_O_3_ nanorods are shown in Fig. S1a,b (see Supplementary Information), which shows the hexagonal and cubic phases respectively. FTIR spectra of as-synthesized Gd_0.925_Eu_0.075_(OH)_3_ and Gd_1.85_Eu_0.15_O_3_ nanophosphors are shown in Fig. S2a,b respectively (see Supplementary Information). In Fig. S2a, the absorption peak at 3400 cm^−1^ is characteristic of hydrogen bonding (hydroxyl stretches) among surface-adsorbed water molecules and the peaks at ~1200 cm^*−*1^ and ~1300 cm^*−*1^ are ascribed to the vibration of NO_3_^*−*^, which originated from the residual NO_3_. The FTIR spectrum of heated nanorods at 800 °C shows no peak around 3400 cm^−1^ due to absence of OH group as shown in Fig. S2b (see Supplementary Information), which suggests that the hydroxide formed (Gd(OH)_3_:Eu^3+^) at 180 °C during hydrothermal process is completely converted into oxide form (Gd_2_O_3_:Eu^3+^) on calcination temperature at 800 °C^30^. The important absorption peak at 748 cm^*−*1^ is assigned to vibration of Gd–O, suggesting that the Gd(OH)_3_:Eu^3+^ nanophosphors has converted to Gd_2_O_3_:Eu^3+^ nanorods after calcinating as shown in Fig. S2 (see Supplementary Information); this result is in good agreement with obtained XRD results. The X-ray photoelectron spectra provide information about the binding states of different atomic species within tens of nanometer of the surface of the material. The elemental analysis of synthesized nanorods was examined by XPS studies. Figure S3 (see Supplementary Information) show the X-ray photoelectron spectrum of Gd_1.85_Eu_0.15_O_3_ nanorods. It reveals the presence of gadolinium (Gd 3d and Gd 4d states), europium (Eu 3d and Eu 4d states) and oxygen (O 1s state) elements in Gd_1.85_Eu_0.15_O_3_ nanorods. The result clearly demonstrates the intensity ratio of Gd/Eu is about more than 11 times and thus justifies the presence of higher amount of Gd as compared to Eu in the host lattice which is also clear from empirical formula of Gd_1.85_Eu_0.15_O_3_. For, better understanding, we have done the XPS of all samples prepared in this series, Gd_2−x_Eu_x_O_3_ (x = 0.05 to 0.5). The intensity ratio of Gd/Eu for all samples are shown in Table TS3 in Supplementary information. The obtained results justify the empirical formula of all samples.

To examine the surface morphology, size and the nanorod formation of as-synthesized nanophosphors, the samples were investigated by scanning electron microscopy (SEM) as well as high resolution transmission electron microscopy (HRTEM). The SEM image of as-synthesized Gd_0.925_Eu_0.075_(OH)_3_ and its magnified view are shown in Fig. 1c,d, respectively. The Gd(OH)_3_:Eu^3+^ sample is composed of a large number of sunflower like superstructures having diameter 1–3 *μ*m. A magnified view of SEM image (Fig. 1d) further reveals that the flower is made up of many thin petals of ~20 nm thickness. The SEM image of as-synthesized Gd_1.85_Eu_0.15_O_3_ is shown in Fig. 1e. The sample with rod like morphology can be confirmed by the magnified SEM image, shown in Fig. 1f. It can be clearly seen that the sample consists of uniform nanorods in high yield (90%) with diameters of about 20 nm and length is in micrometer scale range (~0.6 μm). It is also observed that the samples are straight and the surfaces are very smooth and unifom. The proposed mechanism of the flower-like hydroxide nanostructure formation and its conversion into nanorods like oxide nanostructures have been described in Fig. S4 (see Supplementary Information).

**Figure 2:**
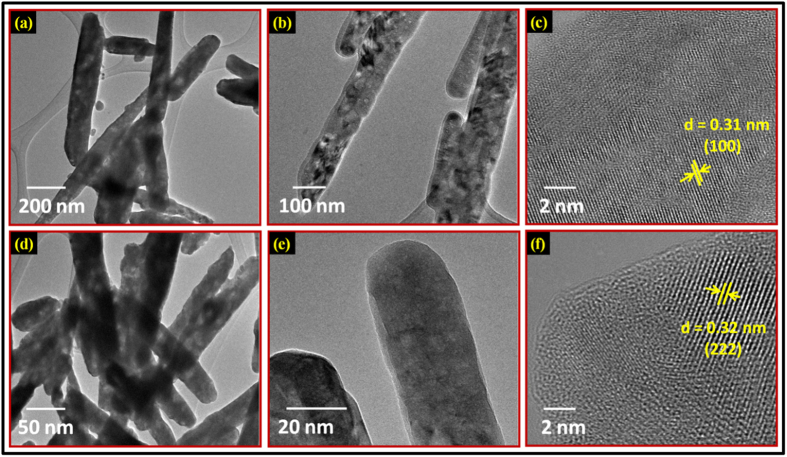
(**a**) TEM image of as-synthesized Gd_0.925_Eu_0.075_(OH)_3_ nanophosphor. **(b)** Magnified view of Fig. (**a**) **(c)** HRTEM image of as-synthesized Gd_0.925_Eu_0.075_(OH)_3_ nanophosphor. **(d)** TEM image of as-synthesized Gd_1.85_Eu_0.15_O_3_ nanorods. **(e)** Magnified view of Fig. (d). **(f)** HRTEM image of as-synthesized Gd_1.85_Eu_0.15_O_3_ nanorods.

The sample was further examined by transmission electron microscope. The TEM micrographs shown in Fig. 2a,b illustrates the morphology of Gd_0.925_Eu_0.075_(OH)_3_ nanophosphors and its magnified view, respectively. The high resolution TEM (HRTEM) image of Gd_0.925_Eu_0.075_(OH)_3_ is shown in Fig. 2c. Figure 2d shows the TEM image of the as-prepared Gd_1.85_Eu_0.15_O_3_ nanorods sample, clearly exhibiting that the sample is entirely composed of relatively uniform nanorods with lengths of 0.6 μm and diameters of about 20 nm, which is consistent with the result shown in the SEM images. The magnified view of Gd_1.85_Eu_0.15_O_3_ nanorods sample is given in Fig. 2e. The morphology of the sample is rod shaped due to the initial nucleation of hexa-hydroxy flower-like phase formation during the intermediate reaction at particular pH value (after hydrothermal process before final calcination at 800 °C for oxide phase formation)^31^. The HRTEM image of Gd_0.925_Eu_0.075_(OH)_3_ and Gd_1.85_Eu_0.15_O_3_ sample are shown in Fig. 2c,f, respectively. The observed fine fringes are associated with the regular crystalline lattice. These results further confirmed that the presence of highly crystalline Gd_1.85_Eu_0.15_O_3_ phase after annealing the Gd_0.925_Eu_0.075_(OH)_3_ nanophosphor sample at optimum temperature, agreeing well with the XRD results. The Gd_0.925_Eu_0.075_(OH)_3_ nanophosphor sample exhibits lattice fringes with interplanar spacing of 0.31 nm, corresponding to the (100) plane of hexagonal Gd_0.925_Eu_0.075_(OH)_3_ crystal (Fig. 2c). The Gd_1.85_Eu_0.15_O_3_ nanorod sample reveals clear lattice fringes with estimated interspacing of 0.32 nm that reflects the (222) plane of Gd_1.85_Eu_0.15_O_3_ with cubic phase (Fig. 2f). The TEM image at another place of sample and selected area electron diffraction (SAED) of as-synthesized Gd_1.85_Eu_0.15_O_3_ nanorods is also shown in Figs S5 and S6, respectively (see Supplementary Information). The electron diffraction pattern (Fig. S6) shows cubically arranged sharp diffraction spots which correspond to the *d* spacing of cubic Gd_1.85_Eu_0.15_O_3_, in agreement with the XRD results.

UV-visible spectra were collected using a high resolution UV-Vis spectrophotometer (Shimadzu, UV-2450) using quartz cells. Spectra were obtained for as-prepared nanophosphor after sonication in distilled water so as to yield homogeneous dispersion. Figure S7 (see Supplementary Information) shows the UV–Vis absorbance spectra of the Gd_1.85_Eu_0.15_O_3_ nanorods sample. A prominent absorption peak at 245 nm is observed in cubic Gd_2_O_3_:Eu^3+^ crystal. The absorption in the UV region is due to the transitions involving extrinsic states such as surface traps or defective states or impurities. The maximum absorption arises due to transitions between the valence to conduction band. Inset of Fig. S7 (see Supplementary Information) shows the enlarged portion of the UV–Vis spectra within the range 350–500 nm.

To understand the thermal decomposition of as-synthesized Gd_2−x_Eu_x_O_3_ (x = 0.05 to 0.5) nanorods, the as-synthesized hydroxide material (Gd_0.925_Eu_0.075_(OH)_3_) was subjected to thermogravimetric analysis (TGA) - differential scanning calorimetry (DSC) investigation in a temperature range 25–900 °C under ambient pressure. Figure S8 (see Supplementary Information) exhibits the TGA analysis of Gd_1.85_Eu_0.15_O_3_ nanorods by taking Gd_0.925_Eu_0.075_(OH)_3_ as a starting material. The DSC curve reveals two endothermic peaks (315 and 432 °C), indicating that the dehydration of the metal hydroxide takes Gd_0.925_Eu_0.075_(OH)_3_ two steps. The balance chemical reaction of entire process is shown in Supplementary Information. It can be observed from TGA curve that there are two major steps of rapid weight loss in the TGA curve at about 284 and 390 °C, accompanying their corresponding endothermic peaks. It indicates that the formation of Gd_2_O_3_:Eu^3+^ nanorods by the annealing of Gd(OH)_3_:Eu^3+^ nanophosphors undergoes two steps. The weight loss for the two steps and the total weight loss are 12%, 11%, and 13.9%, respectively. The two-step dehydration indicates the existence of an intermediate phase other than the precursor Gd(OH)_3_ and the final product Gd_2_O_3_. The obtained result is consistent with earlier reported results^30^,^32^,^33^,^34^. Generally, the photoluminescence intensity of rare-earth hydroxides is much lower than the rare-earth oxides due to the existence of non-radiative relaxation channels provided by high energy vibration of hydroxyl species. Therefore, compared with rare-earth hydroxide precursors, rare-earth oxides often exhibit superior photoluminescence properties and are better prospects for practical applications^35^. These reasons inspired us to consider the thermal transformation from a rare-earth hydroxide flower-like nanostructure into the corresponding oxide nanorods.

The optical characteristics and performances of nanometer- sized phosphor materials are generally dependent on their size and morphologies. Therefore, the effect of morphology on the photoluminescence (PL) intensity of Gd_2_O_3_:Eu^3+^ phosphor was examined to provide a deeper insight of the particle shape of the oxide nanophosphor with high PL efficiency. In order to optimize the photoluminescence intensity and high brightness of the Gd_2−x_Eu_x_O_3_ nanophosphor, the Eu concentration (mol %) in the nanophosphor was varied from x = 0.05 to 0.50. Also, the growth time and temperature were varied from 1 to 6 hrs and from 500 to 1100 °C, respectively. It has been observed that the Eu concentration of x = 0.15, temperature of 800 °C and time of 3 hrs were found to be optimum for the synthesis of nanophosphor with high photoluminescence intensity (see Supplementary Information, Figs S9 and S10) with quantum yield more than 90%. Figure S9 shows an initial steady increase in the PL intensity as the Eu concentration is increased up to x = 0.15; however, beyond this optimum value, the PL intensity began to decrease rapidly and hence quantum yield also decreases. This may be due to luminescence quenching as a result of excess Eu^3+^ ions. It is well established that the Eu doping concentration can affect the distances between Eu ions in the host lattice (Gd_2_O_3_). When the Eu concentration is ~7.5 mol%, the distance between two Eu ions is large, and every Eu^3+^ ion can be regarded as an isolated luminescent centre that independently emits light without any interference. On the other hand, for doping concentrations beyond 7.5 mol%, nearby Eu^3+^ ions can mutually interact by an electric multipolar process due to the shortened distances between Eu^3+^ ions; in this case, the energy transfer rates of Eu^3+^ ions easily exceed the radiative rates. Thus, the absorbed photon energy rapidly migrates among Eu^3+^ ions in the host lattice, decreasing the probability of radiative transitions of Eu^3+^ ions, and even quenching the fluorescence if the excited state gets trapped in an energy sink with a high non-radiative deactivation rate constant. This phenomenon is also known as concentration quenching of fluorescence. Therefore, it is very crucial to choose an appropriate doping concentration to obtain highly efficient photoluminescence when designing lanthanide doped phosphors. After optimizing the Eu concentration, we carried out further experiments to optimize the growth temperature for perfect phase formation. Growth time and growth temperature are important parameters that can improve the overall crystallinity of the phosphor without significantly increasing the size of the nanophosphor, to further improve the PL intensity. Figure S10 shows the dependence of the relative PL peak intensity (emission at 610 nm) on growth temperature as well as growth time. The relative peak intensity and brightness increase as the growth temperature is increased from 500 to 800 °C. However, the PL intensities of the phosphors synthesized at temperatures above 800 °C decrease sharply. This could be due to the creation of secondary oxide phases of gadolinium oxide at high temperatures or the improper diffusion and placement of Eu^3+^ ions at unfavourable gadolinium sites. The other cause may be that the number of radiative recombinations gradually decreases compared to non-radiative recombinations at temperatures above the optimum temperature and that material defects form quenching centres, leading to non-radiative recombination and luminescence quenching. Therefore, the change in emission intensity should be mainly associated with the defects that come from the surface states of phosphors after the recombination of electron–hole pairs. The crystallization of the phosphor improved with increasing growth temperature up to the optimum temperature (800 °C), and defects decreased accordingly as a consequence. Further increases in growth temperature lead to increased defects^4^,^28^,^31^. Figure S11 and S12 (see Supplementary Information) show the photoluminescence excitation (PLE) spectrum of as-synthesized flower-shaped Gd(OH)_3_:Eu^3+^ and nanorod-shaped Gd_2_O_3_:Eu^3+^ nanophosphors at the emission wavelength of 610 nm. The combined PLE spectra are also shown in Fig. S13 (see Supplementary Information). The PLE spectrum (Fig. S12, see Supplementary Information) recorded at 610 nm emission contains a wide band peaking at ~252 nm at room temperature, which is attributed to the transition of the charge transfer state (CTS) of Eu^3+^-O^2−^. This strong and broad band is attributed to the charge transfer absorption from the oxygen ligands to the central gadolinium atoms within the O^2−^ groups. The presence of the Gd_2_O_3_ host band in the excitation spectrum of Eu^3+^ indicates that there exists an efficient energy transfer from Gd_2_O_3_ host to the doped Eu^3+^. The weak shoulder at 276 nm superimposed on the CTS of Eu^3+^ can be assigned to the ^8^S-^6^I transition line of Gd^3+ ^31,32. In addition to the 252 and 276 nm excitations, more other peaks also appeared, as shown in inset of Fig. S12 (see Supplementary Information), which are assigned to the direct excitation of the f-f transitions of the Eu^3+^ ions within its 4f ^6^configuration^33^,^34^,^35^,^36^,^37^.

**Figure 3:**
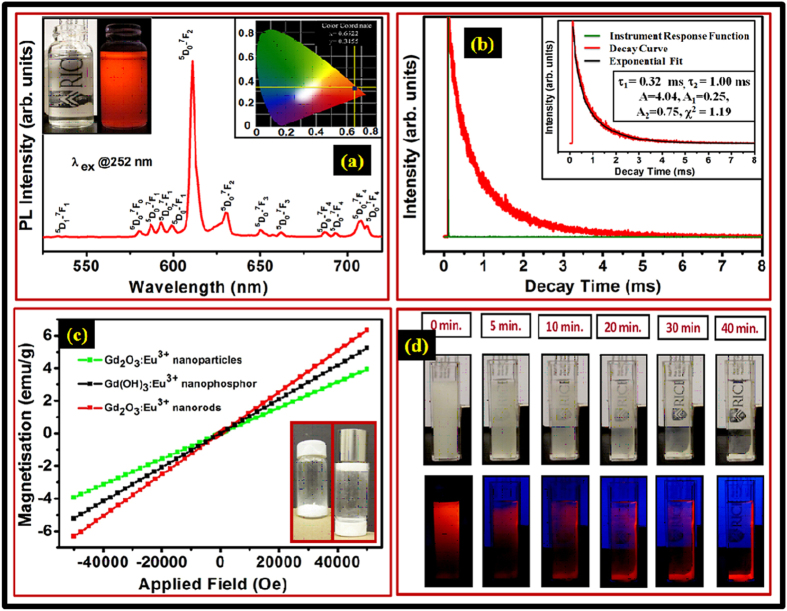
(**a**) PL emission spectrum of as-synthesized Gd_1.85_Eu_0.15_ O_3_ nanorods recorded at 252 nm excitation showing a sharp, intense, hypersensitive red emission peak with maximum at 610 nm (^5^D_0_ – ^7^F_2_) at room temperature; left inset shows typical photographs of as-synthesized Gd_2_O_3_:Eu^3+^ nanorods sample in DI-water under room light as well as a 252 nm UV lamp (a strong red emission of Eu^3+^ is observed under UV excitation) and the right inset shows the color coordinates x = 0.6574 and y = 0.3424. **(b)** TRPL decay profile of as-synthesized Gd_1.85_Eu_0.15_O_3_ nanorods recorded at room temperature while monitoring the emission at 610 nm at an excitation wavelength of 252 nm; inset shows the lifetime data and the parameter generated by the exponential fitting. **(c)** Room-temperature M-H curve of as-synthesized Gd_2_O_3_:Eu^3+^ nanoparticles, Gd(OH)_3_:Eu^3+^ nanophosphor and Gd_2_O_3_:Eu^3+^ nanorods; photographs of luminomagnetic as-synthesized Gd_1.85_Eu_0.15_O_3_ nanorods in glass vials i) without and ii) with an external permanent magnet (≈3000 Oe) are shown in inset. **(d)** Magnetic tracking of luminomagnetic as-synthesized Gd_1.85_Eu_0.15_O_3_ nanorods in an aqueous dispersion showing sequential photographs as a function of time under ambient as well as UV light (252 nm) with external permanent magnet (≈4000 Oe).

The PL emission spectra of as-synthesized Gd_2_O_3_:Eu^3+^ nanoparticles, flower-shaped Gd(OH)_3_:Eu^3+^ nanophosphor and nanorod-shaped Gd_2_O_3_:Eu^3+^ nanophosphor under UV excitation wavelength 252 nm is displayed in Fig. S14 (see Supplementary Information), respectively. In Gd(OH)_3_:Eu^3+^ nanophosphors, no emission peaks were observed. The emission spectrum of Gd_2_O_3_:Eu^3+^ nanorods consists of a series of emission lines at 538, 580, 587, 593, 599, 610, 630, 650, 662, 683, 693, 707 and 712 nm when excited at 252 nm. The PL spectrum recorded at 252 nm excitation shows a sharp intense hypertensive red emission peak at ~610 nm with quantum yield more than 90%, which is an electric-dipole allowed transition and hypersensitive to the environment as shown in Fig. 3a. The left inset of Fig. 3a shows typical photographs of as-synthesized Gd_2_O_3_:Eu^3+^ nanorod sample in DI-water under room light as well as a 252 nm UV lamp (a strong red emission of Eu^3+^ is observed under UV excitation) and the right inset shows the color coordinates x = 0.6574 and y = 0.3424. The emission spectrum also consists of several other prominent peaks centered at 538, 580, 587, 593, 599, 610, 630, 650, 662, 683,693, 707 and 712 nm. These emission lines correspond to transition from ^5^D_0_ - ^7^F_J_ (J = 1, 2, 3, 4) manifolds of Eu^3+^ i.e., ^5^D_1_ → ^7^F_1_ (538 nm), ^5^D_0_ → ^7^F_0_ (580 nm), ^5^D_0_ → ^7^F_1_ (587, 593, 599 nm), ^5^D_0_ → ^7^F_2_ (610, 630 nm), ^5^D_0_ → ^7^F_3_ (650, 662 nm) and ^5^D_0_ → ^7^F_4_ (683,693, 707 and 712 nm). The strongest one is observed at 610 nm. The ^5^D_0_ - ^7^F_1_ (593 nm) transition is magnetic-dipole-allowed with a selection rule, ΔJ = 1, and its intensity is almost independent of the local environment around Eu^3+^ ions. The ^5^D_0_ - ^7^F_2_ transition is the electric dipole transition due to an admixture of opposite parity 4f^n−1^5d states by an odd parity crystal-field component with a selection rule, ΔJ = 2, according to Judd-Oflet theory^3^,^4^,^29^,^38^. Therefore, it is a hypersensitive transition. The number of ^5^D_0_ - ^7^F_J_ (J = 1, 2, 3, 4) emission lines is governed by the selection rules, which depend on the local symmetry of the crystal fields around the sites that the Eu^3+^ ions occupy. According to crystal field analysis and selection rules, the PL emission of the Eu^3+^ ions at the sites with D_2d_ point symmetry should display two lines at 610 and 630 nm for the ^5^D_0_ - ^7^F_2_ transitions, respectively^3^,^38^. These transitions are clearly visible in our spectrum recorded at room temperature using the high resolution spectrometer. These results confirmed that as red luminescence materials, the color purity and brightness of Gd_2_O_3_:Eu^3+^ is superior to that of Gd(OH)_3_:Eu^3+^, and also suggest that as luminescence host, Gd_2_O_3_ is better than Gd_2_(OH_)3_. The –OH group originated from Gd(OH)_3_:Eu^3+^, absorption surface H_2_O and the impurities on the samples surface serve as quenching centers for the luminescent materials, which decrease the intensity of the Gd(OH)_3_:Eu^3+^ nanophosphors^35^. After the annealing process, the Gd(OH)_3_:Eu^3+^ is converted to Gd_2_O_3_:Eu^3+^ and the impurities dramatically decreased, resulting in the increase of the intensity of the calcined Gd_2_O_3_:Eu^3+^ nanorods samples as shown in Fig. S13 (see Supplementary Information). The PL emission spectra of as-synthesized flower-shaped Gd(OH)_3_:Eu^3+^ and nanorod-shaped Gd_2_O_3_:Eu^3+^ nanophosphors under different excitation wavelength is shown in Figs S15 and S16 (see Supplementary Information), respectively, which are all essentially the same differing only in their intensity. The distinct emission lines observed in the PL spectrum (Fig. S16) are due to transitions from excited ^5^D_0_ to the ^7^F_*j*_ (*j* = 0–4) levels of Eu^3+^ ions. An energy level diagram with all possible radiative transitions of Eu^3+^ ions in Gd_2_O_3_ system is also shown in Fig. S17 (see Supplementary Information).

The decay lifetime is an extremely important parameter related to the quality of material and performance, which can be studied using time resolved photoluminescence spectroscopy (TRPL) particularly in applications such as optical displays and bioimaging^3^,^4^. In general, the exciton lifetime depends upon size and shape of the nanophosphor. It is well known that the efficiency of radiative recombination is directly proportional to the decay time of particular transition. The TRPL was recorded by using a single photon counting technique with a microsecond xenon flash lamp as the source of excitation. The luminescence decay profile of the Gd_1.85_Eu_0_._15_O_3_ nanorods is shown in Fig. 3b and the luminescence decay profile with its exponential fit are shown in inset of Fig. 3b. The decay profile was recorded for the Eu^3+^ transition at 610 nm emission (^5^D_0_ - ^7^F_2_ transition of the Eu^3+^ ions in Gd_2_O_3_ host lattice) at 252 nm excitation wavelength at room temperature. The luminescence decay curve of Gd_2_O_3_:Eu^3+^ sample can be well fitted to double-exponential function as shown in equation (1)


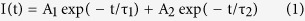


where τ_1_ and τ_2_ are the decay lifetimes of the luminescence, and A_1_ and A_2_ are the weighing parameters. The parameters generated from the fitting routine are listed in the inset of Fig. 3b. For double-exponential decay, the average lifetime, τ_av_, is determined by the following equation (2)^2^,^3^,^4^,^39^,^40^,^41^.


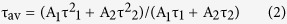


The double component decay attributed to the overlap of closed transition from ^5^D_1_ line (fast decay) and ^5^D_2_ line (slow decay). The average lifetime (τ_av_) of the Gd_1.85_Eu_0_._15_O_3_ nanorods is determined to be 0.93 ms. The results indicate that the synthesized nanophosphor is not only highly suitable for high-contrast bioimaging but also useful for optical display applications.

The magnetic properties were investigated by a superconducting quantum interference device (SQUID). The room temperature magnetization curve (M(H)) for as-synthesized Gd_2_O_3_:Eu^3+^ nanoparticles, Gd(OH)_3_:Eu^3+^ nanophosphor and Gd_2_O_3_:Eu^3+^ nanorods are shown in Fig. 3c. The Gd_2_O_3_:Eu^3+^ nonoparticle was also synthesized using a solid state reaction method. The morphology of the particles is generally spherical having 40 nm particle size. The SEM image of Gd_2_O_3_:Eu^3+^ nanophosphor is shown in Fig. S18 (see Supplementary Information). It is evident that the synthesized luminomagnetic nanorods exhibit typical paramagnetic behavior with high magnetic moment as compared to other samples (nanoparticles and flower-like nanophosphors). The origin of high specific magnetization for rods in comparison to that of nanoparticles is the large amount of exposed surfaces in nanoparticles makes highly canted surface states in spherical shaped nanoparticles. This contributes to a less magnetization in spherical shaped nanoparticles, as it is well cited for ferromagnetic particles^2^. The paramagnetic moment per particle has been calculated using the Langevin function, L (α), (keeping only the first term in the Taylor series expansion of L (α), assuming α is small, which dominates at all practical fields and temperatures; where α is mH/kBT, m is magnetic moment per particle, H is applied magnetic field and kBT is thermal energy). The moment per particle is found to be ≈873 μB (≈67 000 molecules per particle)^4^. This implies that the high paramagnetic moment of Gd^3+^ can be utilized for magnetic contrast imaging due to the possession of higher number of unpaired electrons (seven) in the outer orbital^4^,^12^. One can note from Fig. 3c the Gd_2_O_3_:Eu^3+^ nanorods has approximately 1.2 times higher magnetic moment compared to Gd(OH)_3_:Eu^3+^ nanophosphor. It is also interesting to notice that the observed magnetic moment is quite high as compared to previously reported in literature for other rare-earth based paramagnetic–luminescent nanophosphor^3^,^4^,^5^,^42^,^43^. The behavior of these luminomagnetic nanorods under the influence of permanent magnets both in solid and liquid media has been studied. It is seen that the solid nanorods are stuck on the top of the glass vial when a permanent magnet (~3000 Oe) was placed on the top of the vial (inset of Fig. 3c). We have also carried out the tracking of these luminomagnetic nanorods in the deionized water in a quartz cuvette as depicted in Fig. 3d. A known amount of as-synthesized nanorods was dispersed in deionized water and transferred to quartz cuvette. The cuvette was allowed to stand for 30 minutes after gentle agitation, placing a permanent magnet (~4000 Oe) on one side of the cuvette. Water-dispersed Gd_1.85_Eu_0.15_O_3_ nanorods show bright red fluorescence emission under 252 nm light from a UV lamp, which is demonstrated by spatially defined emission of these nanorods under the influence of an external magnetic field. Nanorods attracted by a permanent magnet are easily visualized by a UV lamp. Sequential photographs were taken after specific time intervals under room light and UV light (254 nm) for comparison (Fig. 3d). It can be seen that the nanorods in water exhibited turbidity thereby blocking the view of the printed Rice logo behind the cuvette. When the permanent magnet was placed on one side of the cuvette, the particles started moving towards the wall of the cuvette where the magnet was placed. At the end of 40 minutes, it was clearly seen that most of the nanorods moved towards the wall containing the magnet, the solution then looking transparent, with the printed logo in the background being completely visible. The slant view of the digital photographs confirm the observations made in the straight view images (see Supplementary Information, Fig. S19). Further, we have also performed DLS studies to confirm the diameter and length distributions of nanorods as well as their dispersibility in two different media; ethanol and DI water as shown in Fig. S20. The obtained results of DLS clearly show that the rod-shaped nanophosphor is highly dispersible in ethanol and DI water with diameter ~20 nm and length ~0.6 μm. Additionally, we have also performed the same experiment for spherical nanoparticles and flower-shaped hydroxides nanophosphor for tracking application. We have taken equal amount of all samples in same amount of DI water for comparative study. The comparative results of magnetic tracking application for spherical nanoparticles, hydroxides flower shaped and rod-shaped oxide nanophosphors are summarized in table TS4. The result clearly shows the minimum time is needed to track nanophosphors after placing the permanent magnet in case of rod-shaped phosphors. These results suggest that the synthesized luminomagnetic nanorods for bioimaging with magnetic tracking ability is the best option when compared to magnetic properties of other samples (nanoparticles and flower like nanophosphors).

**Figure 4:**
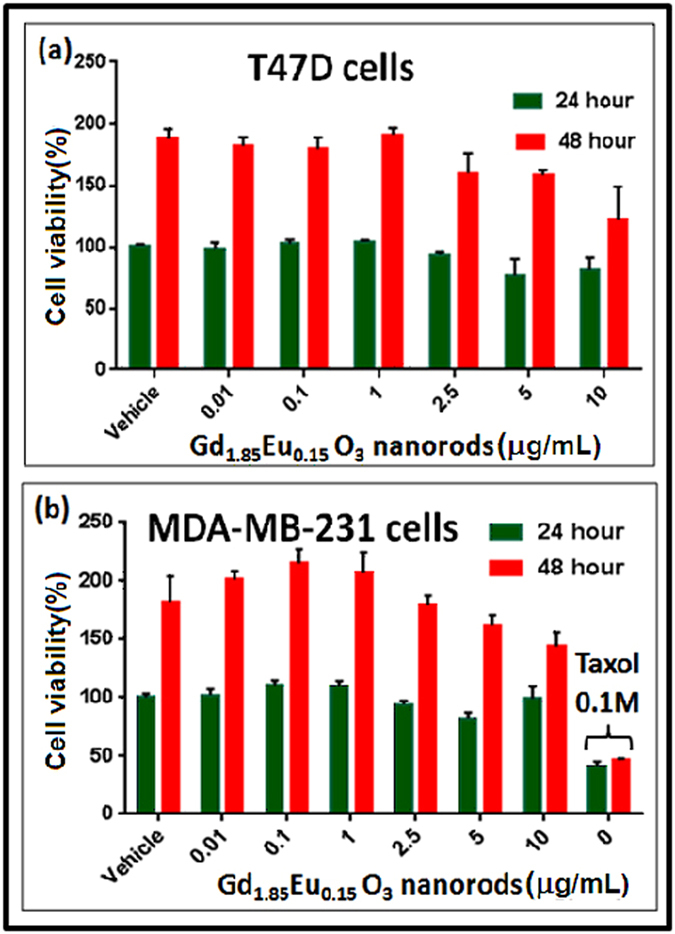
Cell viability assay with human breast cancer cell lines, **(a)** T47D and **(b)** MDA-MB-231 incubated with different concentrations of Gd_1.85_Eu_0.15_O_3_ nanorods. Anticancer drug Taxol was used as a positive control for cytotoxicity assays. The percentage of cells are calculated relative to the dilution medium treated cells at 24 hours.

To examine the biocompatibility of Gd_1.85_Eu_0.15_O_3_ nanorods, we performed cytotoxicity analysis after incubation with Gd_1.85_Eu_0.15_O_3_ nanorods using the standard MTT (3- (4,5-dimethyl-2-yl)-2,5-diphenyltetrazolium bromide) assay at two time points (24 and 48 hours)^2^,^4^,^44^. Two established human breast cancer cell lines (T47D and MDA-MB-231) were used for the MTT assay. Dilution medium (DMEM) treated cells were used as positive control for cell growth and a known anticancer drug Taxol was used as positive control for cytotoxicity. As seen in Fig. 4a,b, no apparent cellular toxicity was observed at up to 5 μg mL^−1^ of Gd_1.85_Eu_0.15_O_3_ nanorods in both cancer cells.

**Figure 5:**
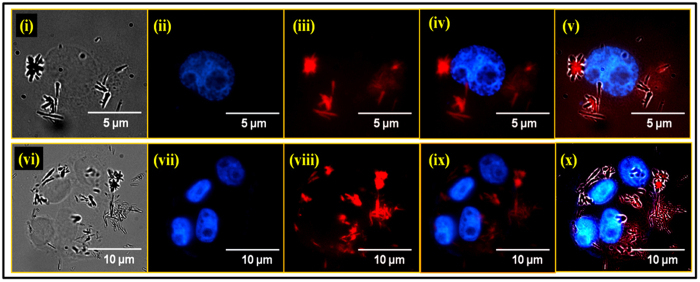
*In vitro* fluorescence microscopy images of T47D cells incubated with Gd_1.85_Eu_0.15_O_3_ nanorods (5 μg mL^−1^) for 4 h. Sequential images of single cell (i–v) and cell cluster (vi–x) show: i) Phase contrast image. ii) Nuclear staining with DAPI. iii) Red fluorescence from Gd_1.85_Eu_0.15_O_3_ nanorods. iv) Overlapped images of blue DAPI and red Gd_1.85_Eu_0.15_O_3_ nanorods (ii and iii). v) Overlap of phase contrast, blue, and red, from (i–iii), respectively. A similar imaging pattern presented for cell cluster with vi) Phase contrast image. vii) Nuclear staining with DAPI. viii) Red fluorescence from Gd_1.85_Eu_0.15_O_3_ nanorods. ix) Overlapped images of blue DAPI and red Gd_1.85_Eu_0.15_O_3_ nanorods (vii and viii). x) Overlap of phase contrast, blue, and red, from (vi–viii), respectively.

To evaluate the cellular imaging potential of Gd_1.85_Eu_0.15_O_3_ nanorods, *in vitro* bioimaging studies have been performed using the T47D (Fig. 5) and MDA-MB-231 (Fig. S21, see Supplementary Information) cells. DAPI (4′,6-diamidino-2-phenylindole) was used to stain the cellular nucleus. Bioimaging of the cell lines using microscopy after overnight incubation with the Gd_1.85_Eu_0.15_O_3_ showed red fluorescence of Gd_1.85_Eu_0.15_O_3_ nanorods mostly in the cytoplasm of the cell (Fig. 5 and S21). The overlap of photoluminescence and phase contrast images confirms the cellular localization of Gd_1.85_Eu_0.15_O_3_ nanorods. To assess the tissue distribution and fluorescence ability *in vivo*, Gd_1.85_Eu_0.15_O_3_ nanorods (80 mg/kg body weight) was injected intraperitoneally in six weeks old C57BL/6J mice and fluorescence intensity was visualized at different time intervals i.e. 1 h, 24 h and 72 h. Gd_1.85_Eu_0.15_O_3_ nanorods injected mice showed higher fluorescence at 600 nm range and fluorescence was seen in all major organs including brain. This indicates that compound can also cross blood brain barrier efficiently. The maximum intensity was found after 1 h of injection which gradually declines and was negligible after 6 h (Fig. S22). These experimental mice did not show any loss in weight and showed normal activity when compared to control animals. In addition, Gd_1.85_Eu_0.15_O_3_ did not cause any adverse effect to the animals as there was no lethality found. This suggests the possibility of using Gd_1.85_Eu_0.15_O_3_ nanorods for both bioimaging and magnetic tracking applications. Moreover, considering the better penetrability of rod shape as well as its dual luminescent and paramagnetic properties, Gd_1.85_Eu_0.15_O_3_ nanorods could be an ultimate choice to be formulated as a next generation promising nanocarrier for the targeted nuclear delivery of pharmacological agents.

## Discussion

A facile and effective method has been developed for the synthesis of ultrafine luminomagnetic Eu-doped Gd_2_O_3_ (Gd_2−x_Eu_x_O_3,_ x = 0.15) nanorods with millisecond photoluminescence lifetime by a base catalyzed hydrothermal method, which can be produced at large-scale with yield more than 90%. This nanophosphor exhibits hypersensitive intense red emission at 610 nm with good brightness, which is an essential feature for high-contrast bioimaging applications, especially to overcome auto fluorescent backgrounds. The efficiency of luminomagnetic nanorods as a biological nanoprobe for high-contrast cell imaging and their cell toxicity were investigated in two human breast cancer cell lines T47D and MDA-MB-231. We have also succesfully performed an *in vivo* experiment to assess the tissue distribution and fluorescence ability. The obtained results clearly demonstrate that the mice did not show any loss in weight and showed normal activity when compared to control animals. Additionally, Gd_1.85_Eu_0.15_O_3_ nanorods did not show any adverse effect to the animals. Moreover, to explore the legitimate role of morphology of the nanostructure, the photoluminescence and paramagnetic characteristic of Gd_2_O_3_:Eu^3+^ nanorods were compared with the spherical nanoparticles of Gd_2_O_3_:Eu^3+^. These nanorods include colloidal stability and optical transparency in water, highly efficient hypersensitive red emission with characteristically sharp spectral lines in visible region, paramagnetic properties and low cellular toxicity. Thus, this approach provides a new highly biocompatible nanoprobe for high-contrast cellular imaging with magnetic tracking capability.

## Methods

### Synthesis of Gd_2−x_Eu_x_O_3_ (x = 0.05 to 0.5) nanorods

In the present investigations, Gd_2−x_Eu_x_O_3_ (x = 0.05 to 0.5) nanorods were synthesized using a facile and effective novel base catalyzed hydrothermal method. The method involves two steps; synthesis of Gd_1−y_Eu_y_(OH)_3_ and calcination of Gd_1−y_Eu_y_(OH)_3_ to form Gd_2−x_Eu_x_O_3_, where x = 2y. The initial amount of raw materials were taken according to empirical formula for final compound Gd_2−x_Eu_x_O_3_ (x = 0.05 to 0.50). We have varied the growth temperature of Gd_1−y_Eu_y_(OH)_3_ form 120 °C to 180 °C, the time from 24 h to 48 h and doping concentration of Eu^3+^ (y = 0.025 to 0.250) in order to find the optimum parameters. The precursor materials such as gadolinium oxide (Gd_2_O_3_, 99.99%), europium oxide (Eu_2_O_3_, 99.99%), nitric acid (HNO_3_, 69% v/v), sodium hydroxide (NaOH, GR grade) and absolute ethanol (C_2_H_5_OH, GR grade) were procured from Sigma-Aldrich. Initially, Gd_2_O_3_ and Eu_2_O_3_ were taken according to the stoichiometric formula and mixed well in a beaker with a 25ml quantity of de-ionized water. Few drops of concentrated HNO_3_ were added to dissolve the oxides and heated at ~100 °C for 1 h under constant stirring to form nitrate solutions. The prepared transparent gadolinium nitrate and europium nitrate solutions were mixed according to stoichiometric formula. Further, 4M NaOH was added to the above rare-earth nitrate solution. The pH of the final solution was monitored and maintained at ~13. This is one of the crucial parameter to obtain high aspect ratio nanorods. We changed the pH value from 8 to 13 and the highest aspect ratio was achieved in pH ~ 13. Subsequently, the obtained solution was mixed with the absolute ethanol (C_2_H_5_OH, GR grade) with the volume ratio, V_solution_:V_ethanol_ = 1:2. Ethanol was used to control uniform growth during continuous stirring of the mixture. The mixed solution was transferred into100 mL autoclavable tubes, sealed and heated at 180 °C for 24 h. The solution was continuously stirred during the hydrothermal treatment. Once the reaction was completed, the autoclave was allowed reach room temperature and the white solid product (Gd_1−y_Eu_y_(OH)_3_) was filtered and washed with distilled water and absolute ethanol for five to six times, respectively. The resulting product was fine white powder. After the dehydration of Gd_1−y_Eu_y_(OH)_3_, in order to obtain Gd_2−x_Eu_x_O_3_ nanorods with high brightness, we have varied the calcination temperature (500 to 1100 °C) and time (1 to 6 h). The optimum condition for Gd_2−x_Eu_x_O_3_ nanorods were obtained through calcination at 800 °C in air for 3 h. In this method, the yield of nanorod was more than 90% with uniform morphology, strong luminescence and high degree of homogeneity. The flow chart for the synthesis of different phases of Eu^3+^ activated flower-shaped gadolinium oxide Gd(OH)_3_:Eu^3+^ nanophosphor and nanorod-shaped Gd_2_O_3_:Eu^3+^ nanophosphors by hydrothermal method is shown in Scheme S1 (see Supplementary Information).

### Characterization of Gd_2−x_Eu_x_O_3_ nanorods

As-synthesized flower-shaped nanophosphors and nanorods were thoroughly characterized using a number of different techniques including powder X-ray diffraction (XRD, Rigaku: MiniFlex, CuKα_1_; λ = 1.5406 Å, angle 10 to 80° at a scanning rate of 2° /min), Raman (Renishaw InVia, 633 nm He-Ne laser), Fourier transform infrared spectroscopy (FTIR, Nicolet FTIR Microscope with an MCT/A detector), X-ray photoelectron spectroscopy (XPS, Perkin Elmer, Model No. PHI1257, a hemispherical electron energy analyzer using non-monochromatized Al (Kα) source (1486.6 eV) with a base pressure of 4 × 10^−10^ Torr at room temperature), scanning electron microscopy (SEM, LEO, Model No. 440), high resolution TEM (HRTEM, Technai, Model No. G20-twin, 200 kV with super twin lenses having point and line resolutions of 0.144 nm and 0.232 nm, respectively) equipped with energy dispersive X-ray analysis (EDAX), Thermal gravimetric analysis and differential scanning calorimetry (TGA, SDT 2960 Simultaneous DSC-TGA, TA Instrument), UV-visible (Shimadzu, Model No. UV-2450), photoluminescence (PL, Edinburgh, Model No. FLSP-920, xenon flash lamp was used as a source of excitation) spectroscopy, time-resolved spectroscopy (TRPL, time correlated single photon counting technique with instrument Edinburgh, Model No. FLSP-920, xenon flash lamp which is equipped with steady state and time-resolved luminescence spectrometer, using a microsecond xenon flash lamp as the source of excitation). In order to estimate the absolute luminescence quantum efficiency of the Gd_1.85_Eu_0.15_O_3_ nanorods, an integrating sphere equipped with an Edinburgh spectrometer (Model FLS920) instrument has been used for measuring the integrated fraction of luminous flux and radiant flux with the standard method. The super conducting quantum interference device (SQUID, Quantum design, SQUID Magnet, MPMS) magnetometric measurements were performed to explore magnetic property of spherical-shaped Gd_1.85_Eu_0.15_O_3_ nanoparticle, rod-shaped Gd_1.85_Eu_0.15_O_3_ nanophosphor and flower-shaped Gd_0.925_Eu_0.075_(OH)_3_.

### Biocompatibility

We used two breast cancer cell lines T47D and MDA-MB-231 for bioimaging and cytotoxicity studies. While, T47D cell is an estrogen receptor alpha (ERα) positive moderately aggressive breast cancer cell line, MDA-MB-231 cell belongs to the triple negative breast cancer subtype with very aggressive cancer metastatic properties. Both these cell lines used extensively in cancer research particularly in hormonal modulation and metastatic breast cancer studies in mice. Thus, the characterization and experimental use of both these cell lines have been extensively reported by several researchers including the present authors^4^,^45^,^46^,^47^,^48^. Thus we used these two established breast cancer cell lines for cytotoxicity and bioimaging studies. The cells were cultured and maintained in DMEM high glucose medium (Invitrogen) containing 4.5 g L^−1^ D-glucose, 4 mM L-glutamine, and 110 mg L^−1^ sodium pyruvate, supplemented with 10% fetal bovine serum (FBS), 100 IU mL^−1^ penicillin and 100 μg mL^−1^ streptomycin in a humidified incubator at 37 °C with 5% CO_2_. Each well of 96 cells culture plate was plated with 4 × 10^3^ cells in100 μL culture medium and incubated overnight. Next day, Gd_1.85_Eu_0.15_O_3_nanorods or Gd_2_O_3_:Eu^3+^ nanoparticles suspended in cell culture medium were added to each well in different concentration (ranging from 0.01–10 μg mL^−1^) in triplicate. Control cells were treated with equal volume of dilution medium (DMEM). After 24 hrs and 48 hrs of incubation, the medium was removed, and the cells were washed gently with 500 μL warm, sterile phosphate buffer solution (PBS). To each well, 200 μL MTT reagent (0.5 mg mL^−1^ in medium) was added and returned to the incubator for 4 hrs. After incubation, medium with MTT reagent was replaced with 200 μL of DMSO. The cells were subsequently incubated for 5 minutes and the optical density of solubilized formazan salts was assessed at 570 nm in a Tecan Infinite M200 microplate reader (Mannedorf, Switzerland).

### *In vitro* Bioimaging

For bioimaging, 1 × 10^4^ cells were plated in each well of a 4 well sterile chamber slide (Nunc, USA) with 500 μL culture medium described above. After overnight culture, 5 μg mL^−1^ Gd_1.85_Eu_0.15_O_3_ nanorods or Gd_2_O_3_:Eu^3+^ nanoparticles was added to the culture medium and incubated in regular cell culture conditions. After both 4 and 24 hours of culture, the medium with Gd_1.85_Eu_0.15_O_3_ nanorods or Gd_2_O_3_:Eu^3+^ nanoparticles was removed from the cells and washed two times with 1 mL PBS. Cells were fixed using 1% paraformaldehyde and mounted with Vectashield antifade mountingmedia with DAPI (Vector Laboratories, Inc., CA). Cellular imaging was done using a Nikon Eclipse 90i microscope equipped with the Cool SNaP HQ2 CCD camera (Photometrics, AZ).

### Animal ethics statement

All mice experiments were approved by animal ethics committee of National Institute of Immunology (IAEC No. 276/11) and conducted in accordance with the Horizontal Legislation on the protection of animals used for scientific purposes, EU directive 2010/63/EU.

### *In vivo* Bioimaging

Whole body imaging was performed using Kodak *in vivo* FX PRO (Kodak, Rochester, NY, USA) employing Carestream Molecular Imaging software version 5.0.2.3.0 (Carestream Health Inc., Rochester, NY, USA). Six weeks old, C57BL/6J mice were used. The Control and Gd_1.85_Eu_0.15_O_3_ nanorods injected C57BL/6J mice were anesthetized with a combination of xylazine (10 mg kg^−1^) and ketamine (75 mg kg^−1^) administered intraperitoneally. Following anesthesia, mice were injected with Gd_1.85_Eu_0.15_O_3_nanorods intraperitoneally and images were acquired at different time intervals such as 5, 15, 30 min, 1 h, 2 h, 4 h, 6h, 24 h and 72 h. Fluorescent images of whole body along with their controls were acquired in multi-wavelength mode using the following settings: exposure type–standard, exposure time - 5 min, excitation filter - 495 nm, emission filter - 600 nm, f-stop - 4.11, FOV - 160 mm.

## Additional Information

**How to cite this article**: Gupta, B. K. *et al.* Bifunctional Luminomagnetic Rare-Earth Nanorods for High-Contrast Bioimaging Nanoprobes. *Sci. Rep.* **6**, 32401; doi: 10.1038/srep32401 (2016).

## Supplementary Material

Supplementary Information
