# Supplementary material for: Bifunctional Luminomagnetic Rare-Earth Nanorods for High-Contrast Bioimaging Nanoprobes
Source: Sci Rep. 2016 Sep 2;6:32401. doi: 10.1038/srep32401 (PMC5009349; doi:10.1038/srep32401)
Supplement: Supplementary Information [file srep32401-s1.pdf]

Supplementary Information

Bifunctional Luminomagnetic Rare-Earth Nanorods for High-Contrast Bioimaging Nanoprobes

Bipin Kumar Gupta*[,1], Satbir Singh[1,2], Pawan Kumar[1,2], Yean Lee[3], Garima Kedawat[4], Tharangattu N. Narayanan[5], Sajna Antony Vithayathil[6], Liehui Ge[3], Xiaobo Zhan[3], Sarika Gupta[7], Angel A. Martí[8], Robert Vajtai[3], Pulickel M. Ajayan[3] and Benny Abraham Kaipparettu*[,6,9]

[1]Luminescent Materials and Devices Group, Materials Physics and Engineering Division, CSIR-National Physical Laboratory, Dr K S Krishnan Road, New Delhi, 110012, India, [2]Academy of Scientific and Innovative Research (AcSIR), CSIR-National Physical Laboratory Campus, Dr K S Krishnan Road, New Delhi 110012, India, [3]Department of Material Science and Nano Engineering Rice University, Houston, TX 77005, USA, [4]Department of Physics, Kalindi College, University of Delhi, New Delhi, 110008, India, [5]TIFR- Center for Interdisciplinary sciences, Tata Institute fundamental research, Hydrabad-500075, India, [6]Department of Molecular and Human Genetics & [9]Dan L. Duncan Cancer Center, Baylor College of Medicine, Houston, TX 77030, USA, [7]National Institute of Immunology, Aruna Aseaf Ali Marg, J. N. U. Complex, New Delhi-110067, India, [8.]Department of Chemistry and Bioengineering, Rice University, Houston, Texas 77005, USA.

*E-mail: bipinbhu@yahoo.com (B.K.G.) and kaippare@bcm.edu (B.A. Kaipparettu)

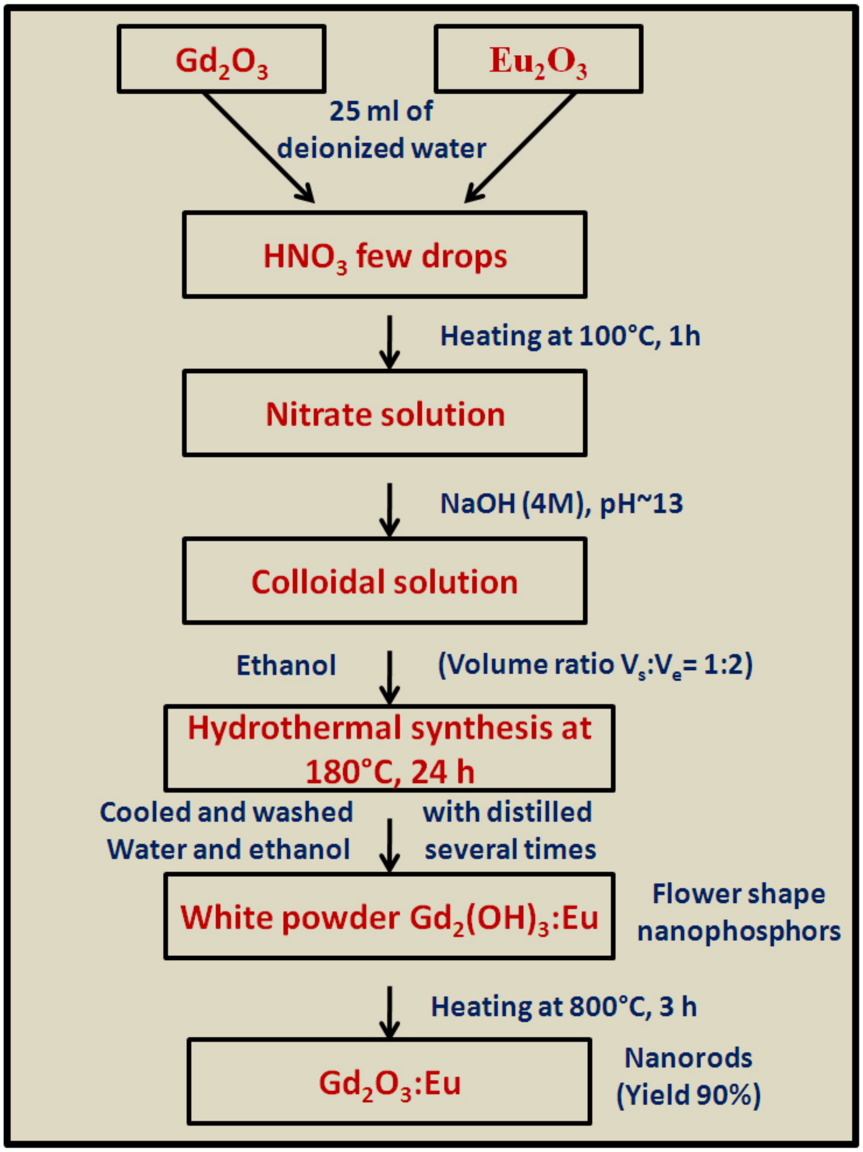

Scheme S1. The flow chart for the synthesis of different phases of Eu^{3+} doped gadolinium oxide (flower- shaped $Gd(OH)_3$ nanophosphors and nanorods-shaped Gd_2O_3 nanophosphors) by hydrothermal method.

The balance chemical reactions of entire process are as follows:

$$Gd_{1-y}Eu_y(NO_3)_3 + 3NaOH \longrightarrow Gd_{1-y}Eu_y(OH)_3 + 3NaNO_3$$

$$Gd_{1-y}Eu_y\,(OH)_3 \longrightarrow Gd_{1-y}Eu_yOOH + H_2O$$

$$2Gd_{1-y}Eu_yOOH \longrightarrow Gd_{2-x}Eu_xO_3 + H_2O,\ 2y=x$$

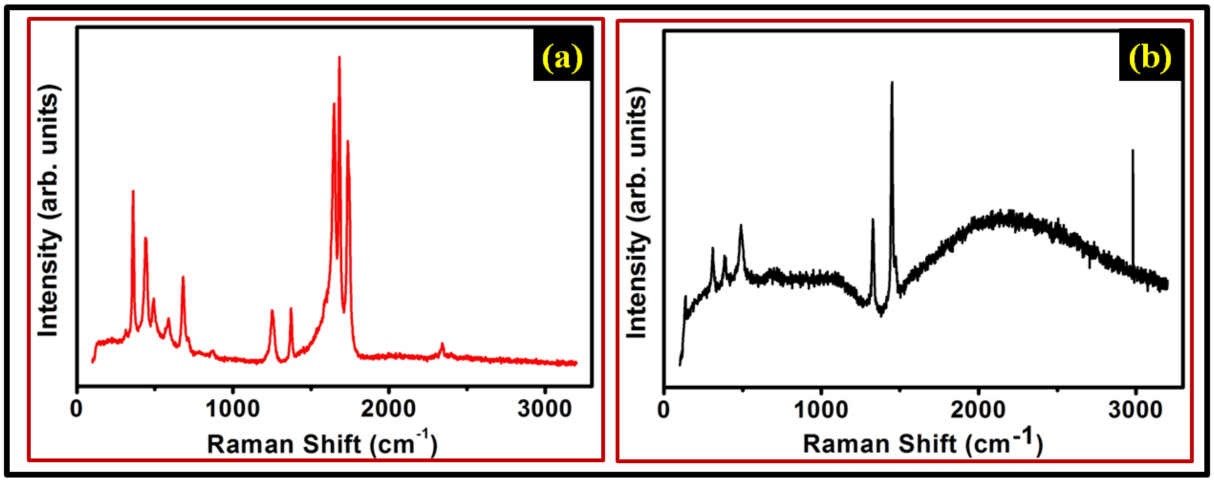

Figure S1. Raman spectrum of as-synthesized (a) flower-shaped $Gd_{0.925}$ $(OH)_3Eu_{0.075}^{3+}$ nanophosphor and (b) nanorods-shaped $Gd_{1.85}Eu_{0.15}O_3$ nanophosphor.

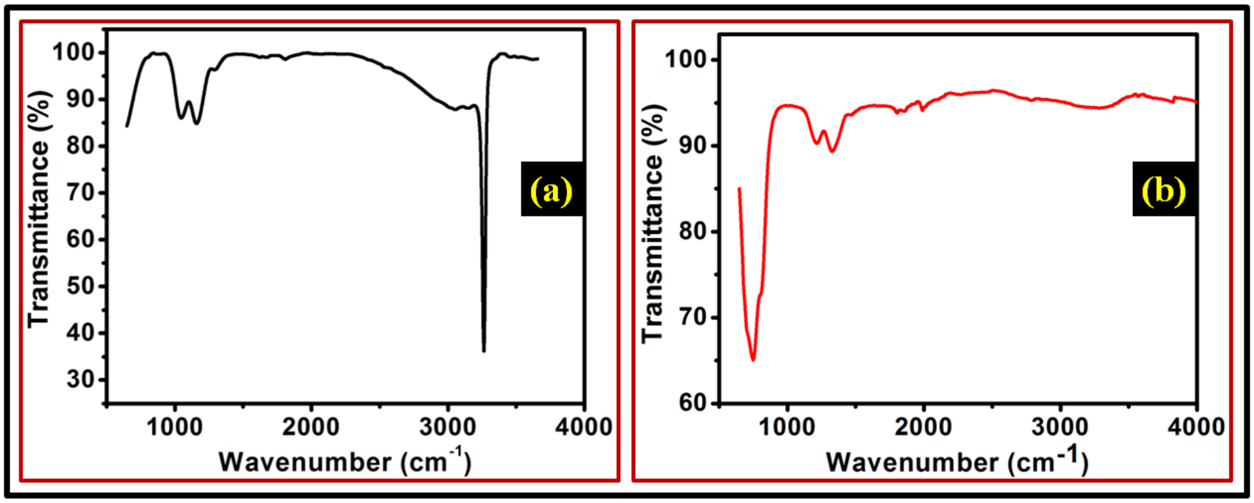

Figure S2. FTIR spectrum of as-synthesized (a) flower-shaped $Gd_{0.925}$ $(OH)_3Eu_{0.075}^{3+}$ nanophosphor and (b) nanorods-shaped $Gd_{1.85}Eu_{0.15}O_3$ nanophosphor.

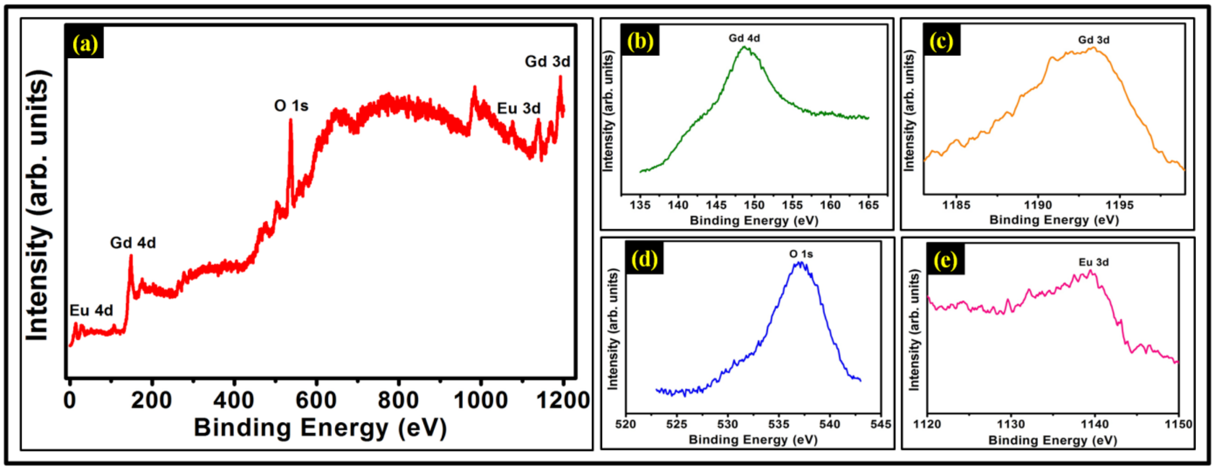

Figure S3. (a) XPS survey scan of as-synthesized $Gd_{1.85}Eu_{0.15}O_3$ nanorods, core level spectra of (b) Gd 4d, (c) Gd 3d, (d) O 1s and (e) Eu 3d elements.

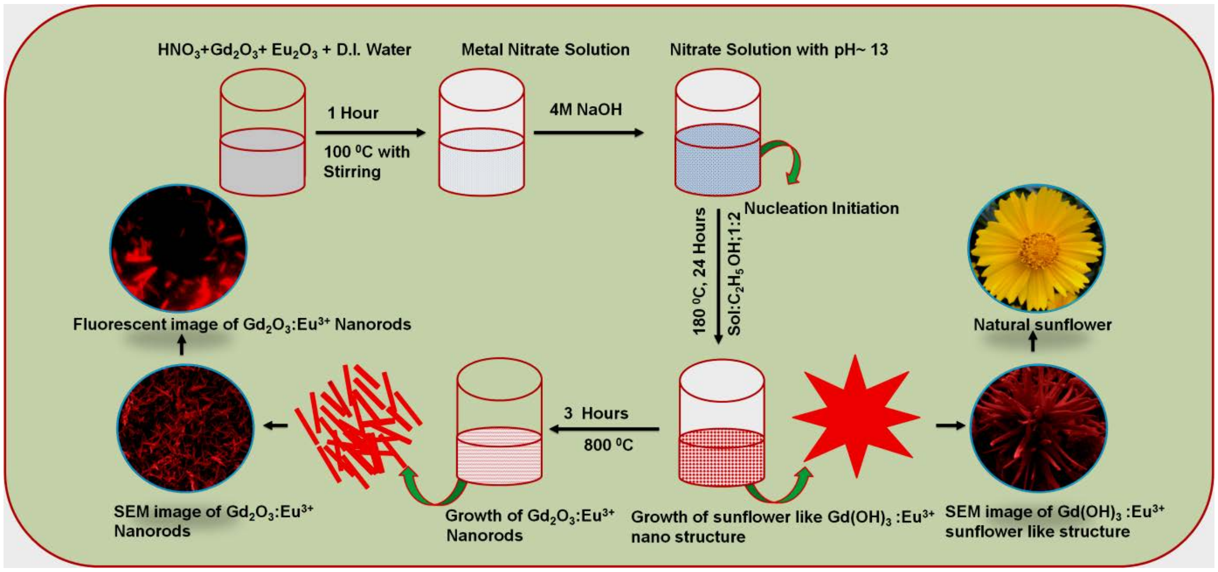

Figure S4. Schematic of proposed mechanism for the growth $Gd_2O_3:Eu^{3+}$ nanorods.

Proposed mechanism for the growth $Gd_2O_3:Eu^{3+}$ nanorods: A plausible mechanism for nucleation of the nanorod like structure depends upon the pH value , growth temperature, doping concentration of the metal complex precursor. This method basically involves two steps: (a) synthesis of sunflower like $Gd_{1-y}Eu_y(OH)_3$ and (b) calcination of sunflower like $Gd_{1-y}Eu_y(OH)_3$ to form $Gd_{2-x}Eu_xO_3$ nanorod (2y=x). Initially, the Gd_2O_3 and Eu_2O_3 were taken

according to the stoichiometric formula and converted into metal nitrate solutions. Further, the pH of the final solution was monitored and maintained at ~ 13 with the addition of NaOH. Because at different pH, the aspect ratio of the converted metal hydroxide nanostructure from metal nitrate is different. The lower pH (<10) initiates the higher diameter (low aspect ratio) nanorods due to availability of isolated nuclei centers in metal precursor. Further, at higher pH (>10) the aspect ratio of the hydroxide nanostructure increases. The sunflower like $Gd_{0.925}Eu_{0.075}(OH)_3$ nanostructure originated due to co-effect of initial nuclei aggregation and crystal growth at higher pH~13 at 180ºC for 24 h duration. It is similar to the growth of natural sunflower structure which initially grows at center with combination of several co-nuclei centers and ultimately converted into sunflower structure as shown in proposed schematic S4. Furthermore, after heating at higher temperature (800 ºC), the synthesized $Gd_{0.925}Eu_{0.075}(OH)_3$ sunflower like structure converted into $Gd_{1.85}Eu_{0.15}O_3$ nanorod like structure because growth of $Gd_{1.85}Eu_{0.15}O_3$ is usually higher along the maximum anisotropic plane direction, as the growth along this plane is faster than any other direction.

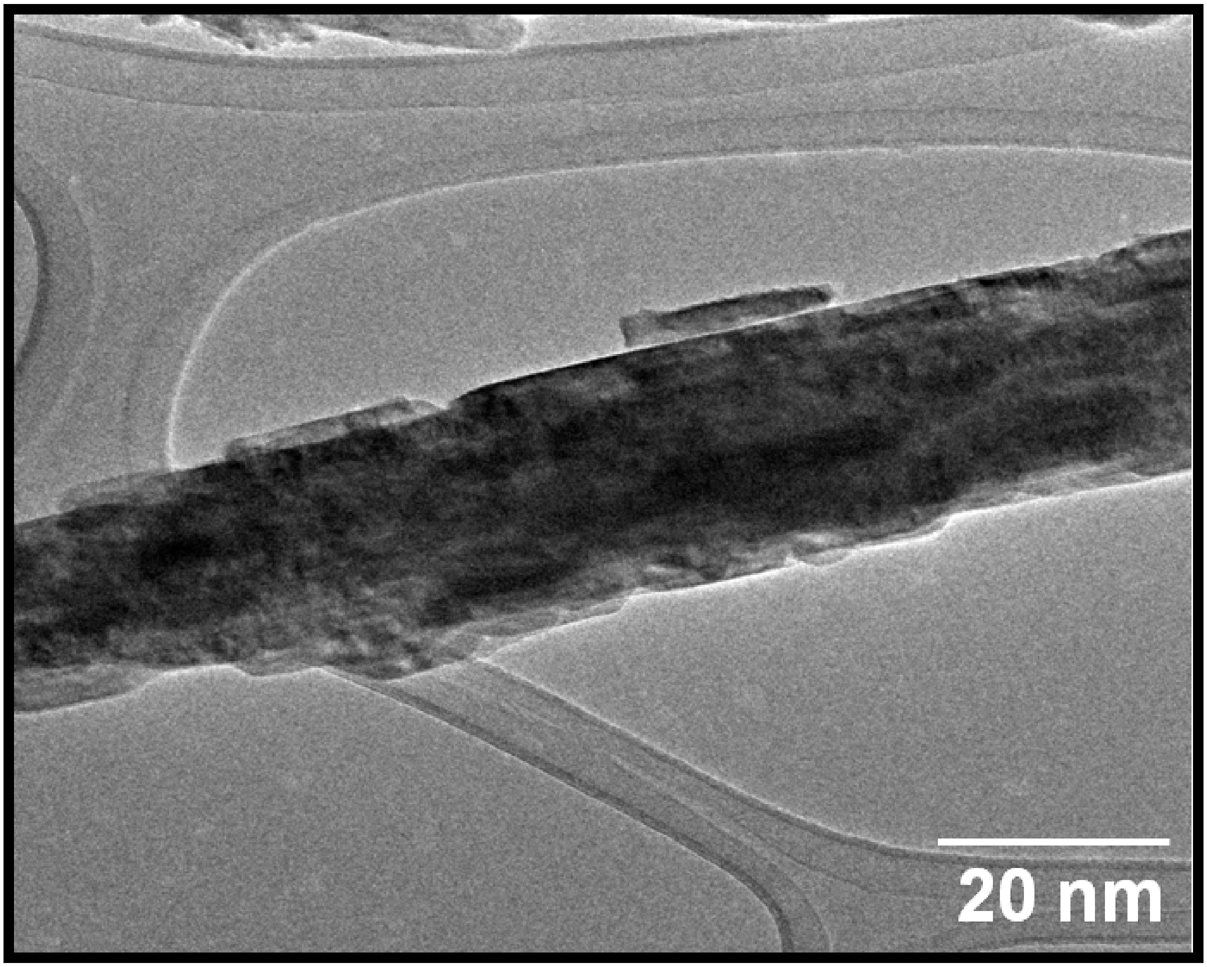

Figure S5. TEM image of $Gd_{1.85}Eu_{0.15}O_3$ nanorods.

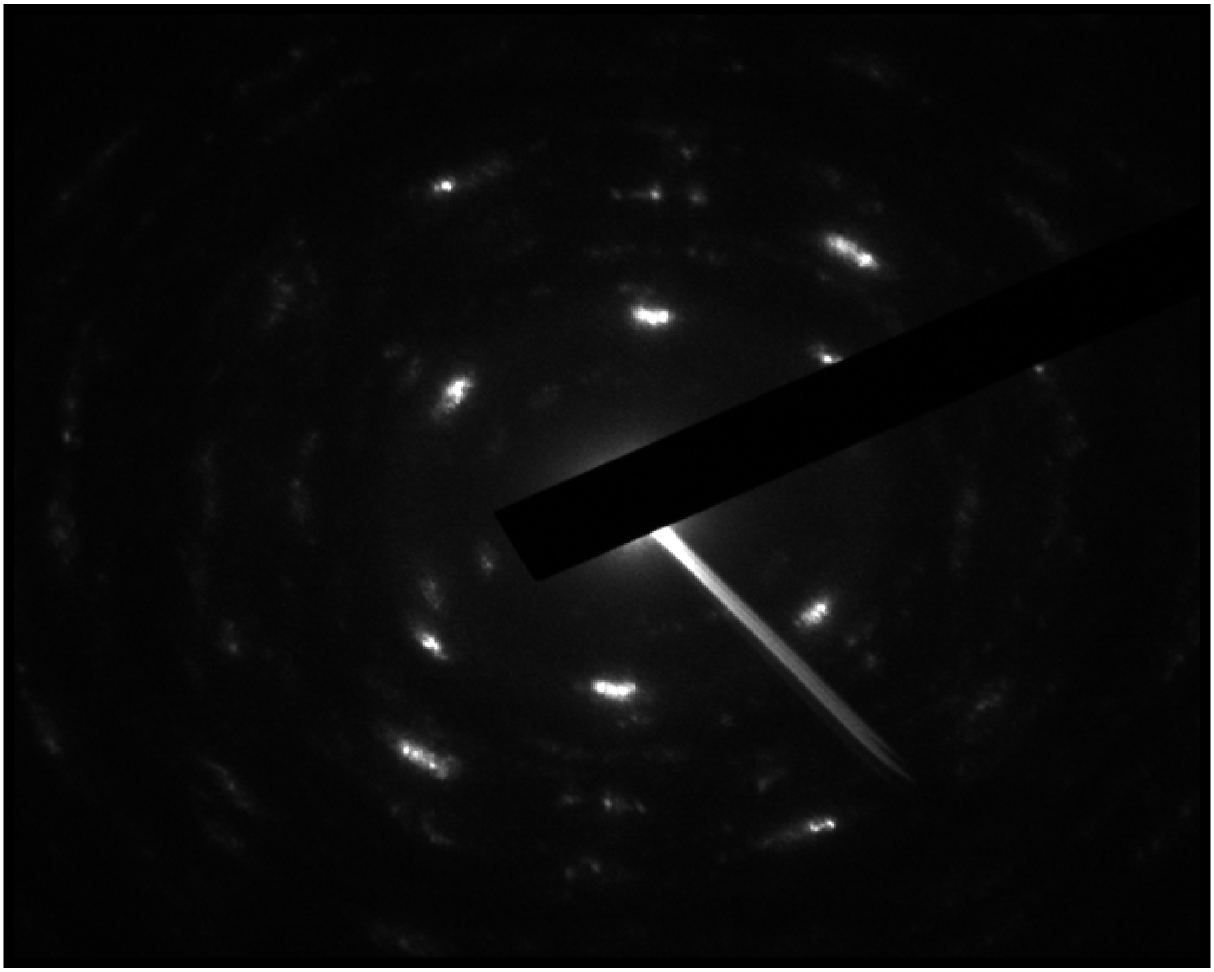

Figure S6. SAED pattern of $Gd_{1.85}Eu_{0.15}O_3$ nanorods.

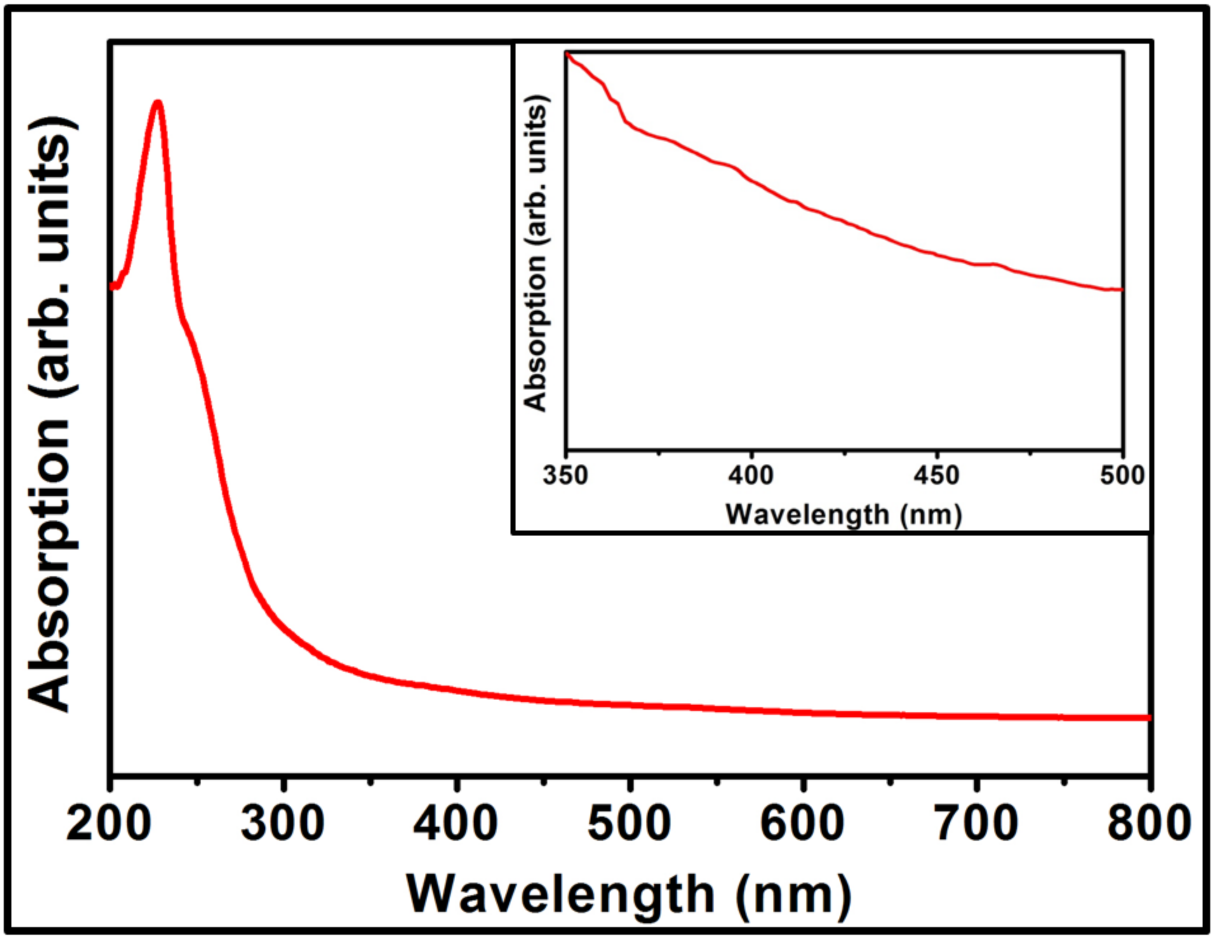

Figure S7. UV-Vis absorption spectrum of $Gd_{1.85}Eu_{0.15}O_3$ nanorods and inset shows the enlarge view of spectrum in the wavelength range 350 – 500 nm.

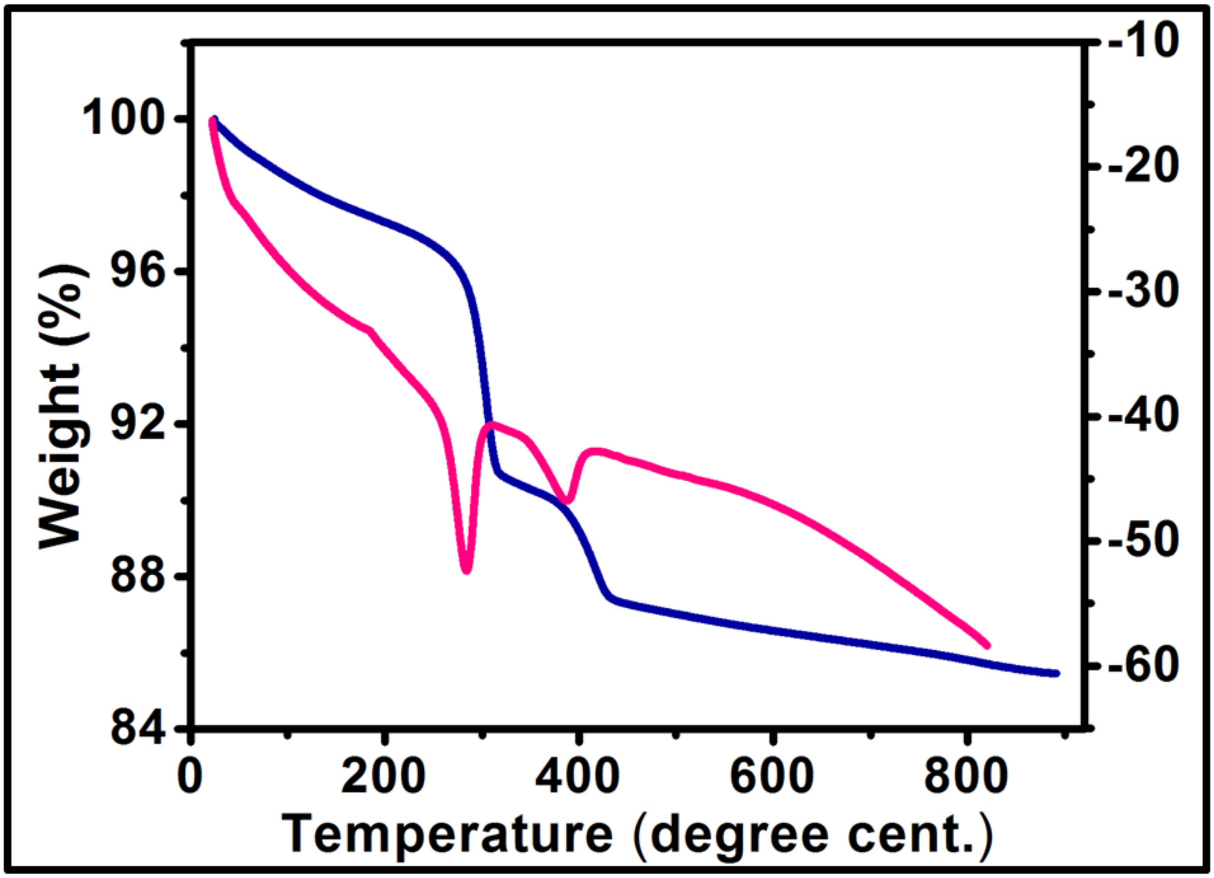

Figure S8.TGA and DSC spectra of $Gd_{1.85}Eu_{0.15}O_3$ nanorods.

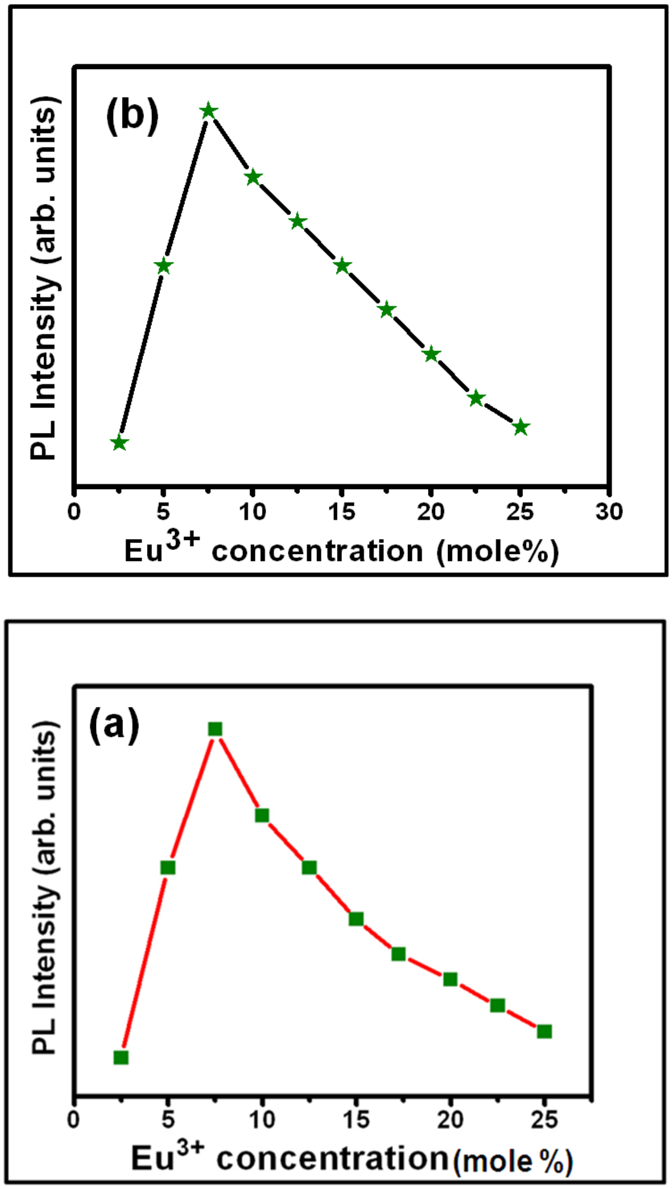

Figure S9.Variation of PL intensity with Eu^{3+} concentration (mole %) in the synthesis of (a) $Gd_{1-y}Eu_y(OH)_3$ (y = 0.025 to 0.25) nanophosphor (black solid line with green star points) and (b) $Gd_{2-x}Eu_xO_3$ (x = 0.05 to 0.5) nanorods (red solid line with green square points).

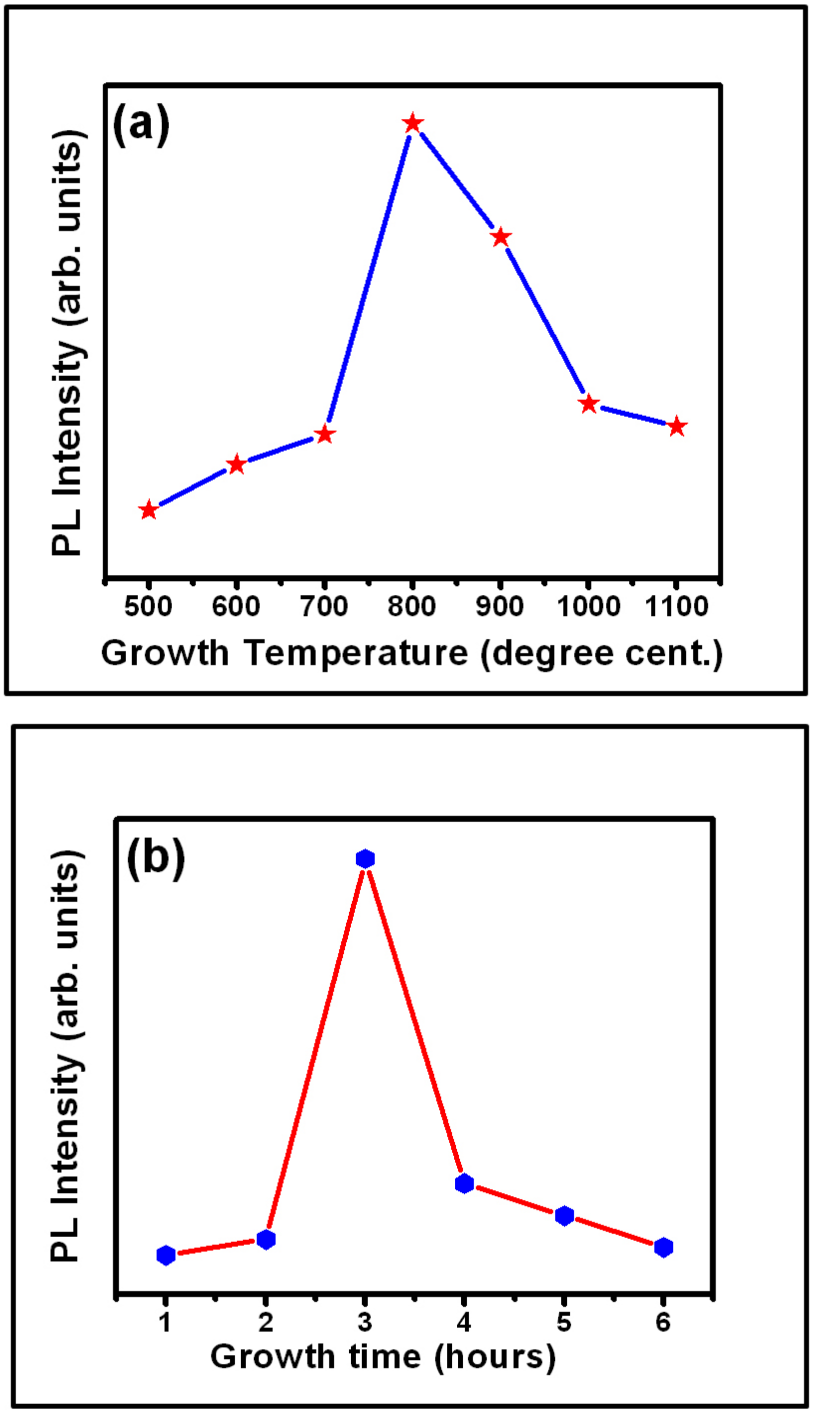

Figure S10. Variation of PL intensity with effect of (a) Growth temperature and (b) Growth time on Eu^{3+} for the synthesis of $Gd_{2-x}Eu_xO_3$ (x = 0.05 to 0.5) nanorods.

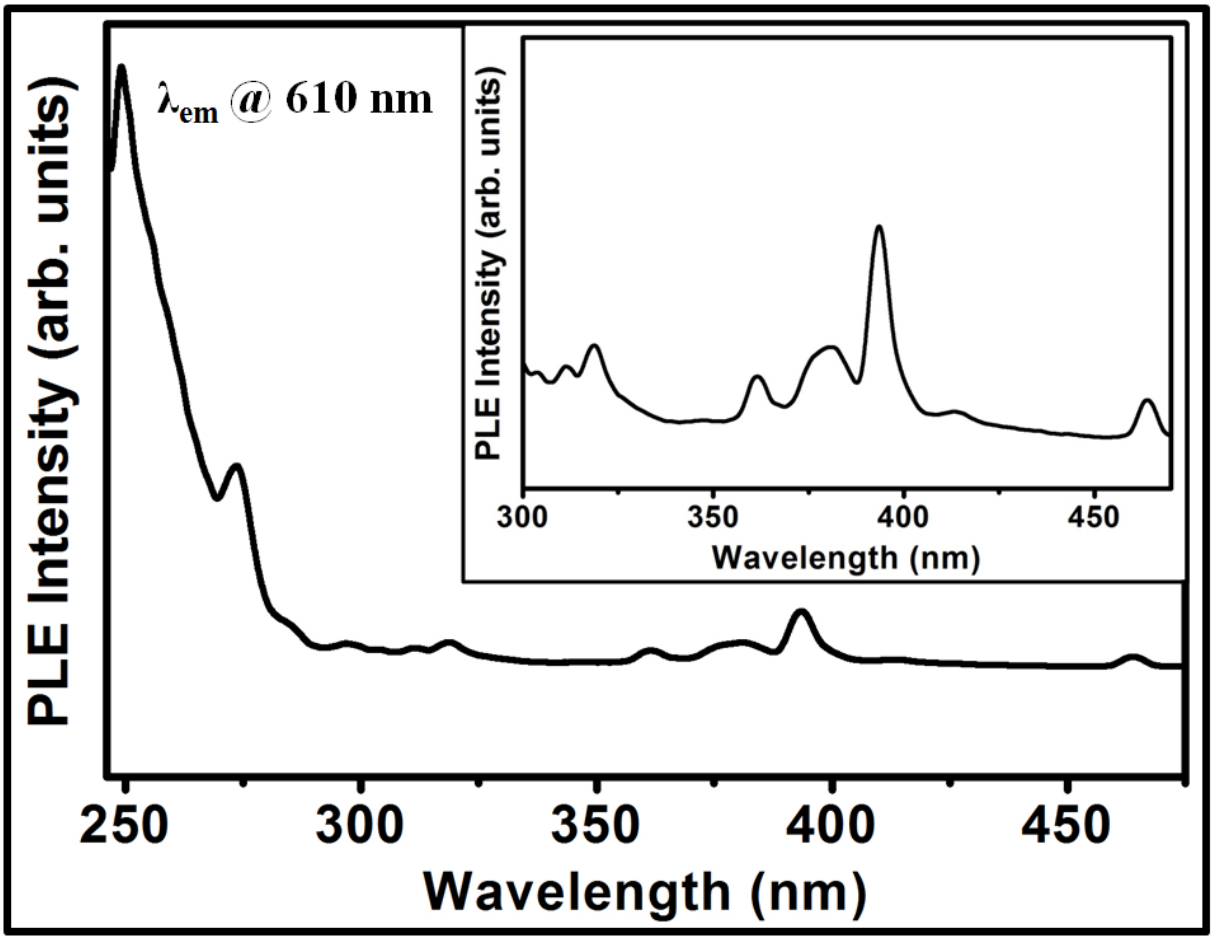

Figure S11. PLE excitation spectrum of $Gd_{0.925}Eu_{0.075}(OH)_3$ nanophosphors under the emission wavelength 610 nm and inset shows the enlarge view of spectrum in the wavelength range 350 – 475 nm.

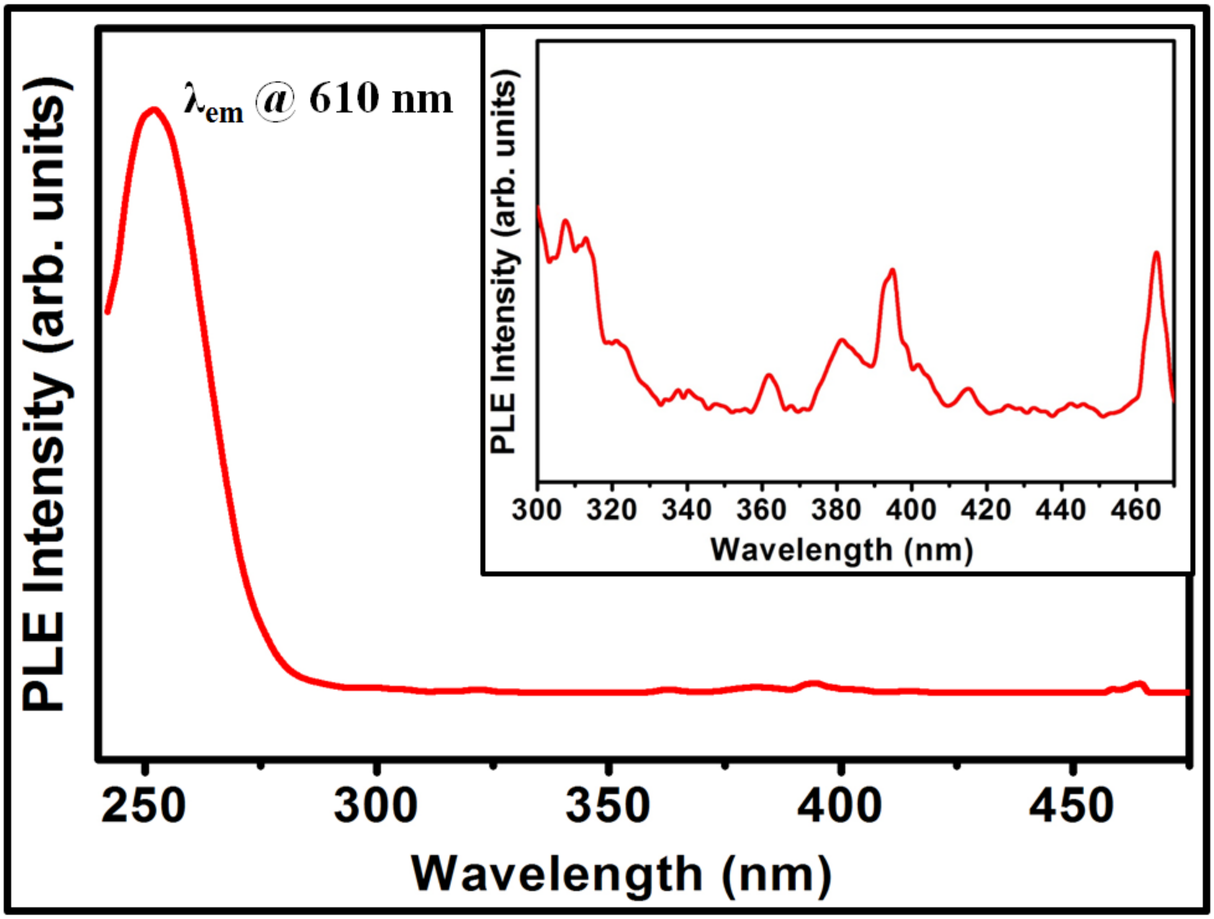

Figure S12. PLE excitation spectrum of $Gd_{1.85}Eu_{0.15}O_3$ nanorods under the emission wavelength 610 nm and inset shows the enlarge view of spectrum in the wavelength range 300 – 470 nm.

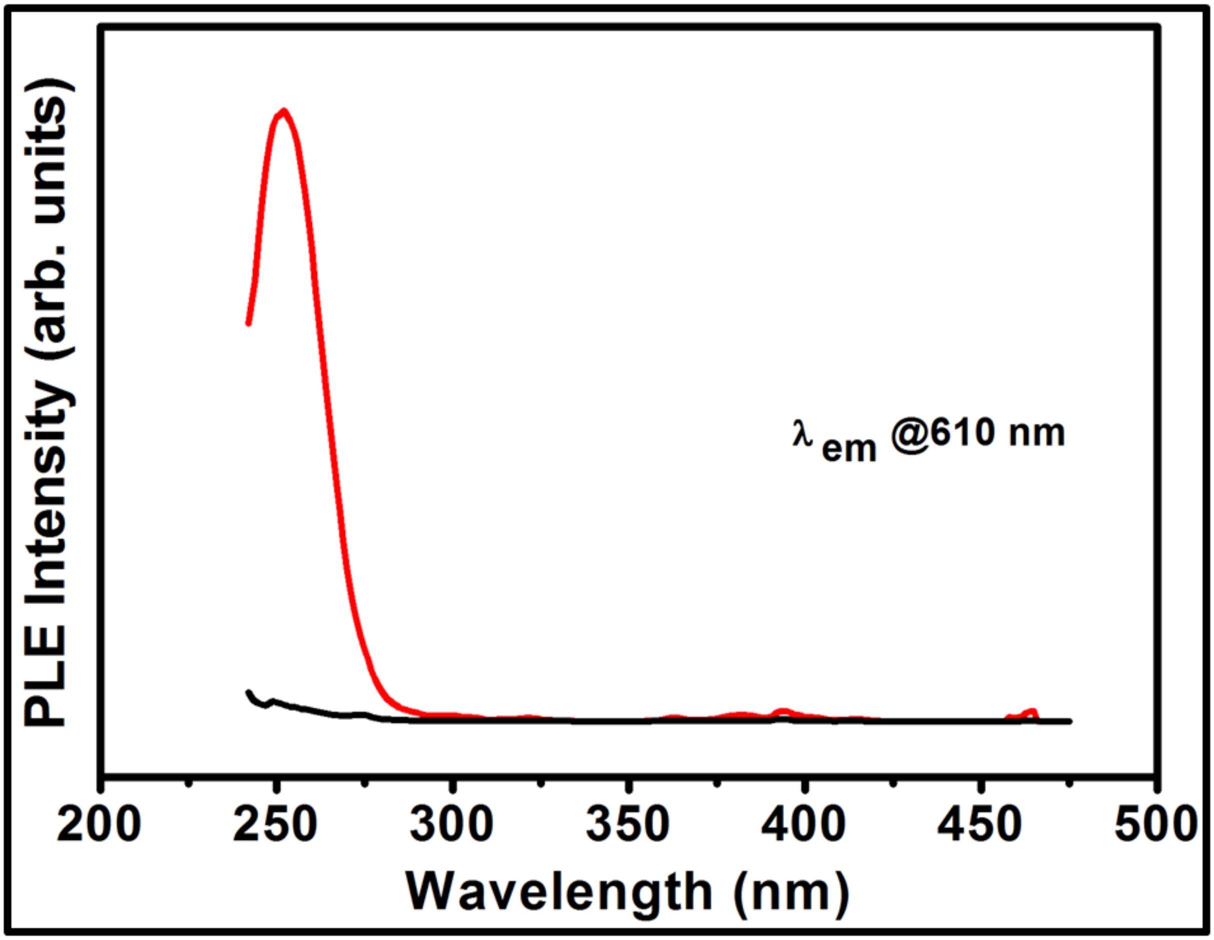

Figure S13. Combined effect of PLE excitation spectra of $Gd_{0.925}Eu_{0.075}(OH)_3$ nanophosphor (black solid line) and $Gd_{1.85}Eu_{0.15}O_3$ nanorods (red solid line) under the emission wavelength 610 nm.

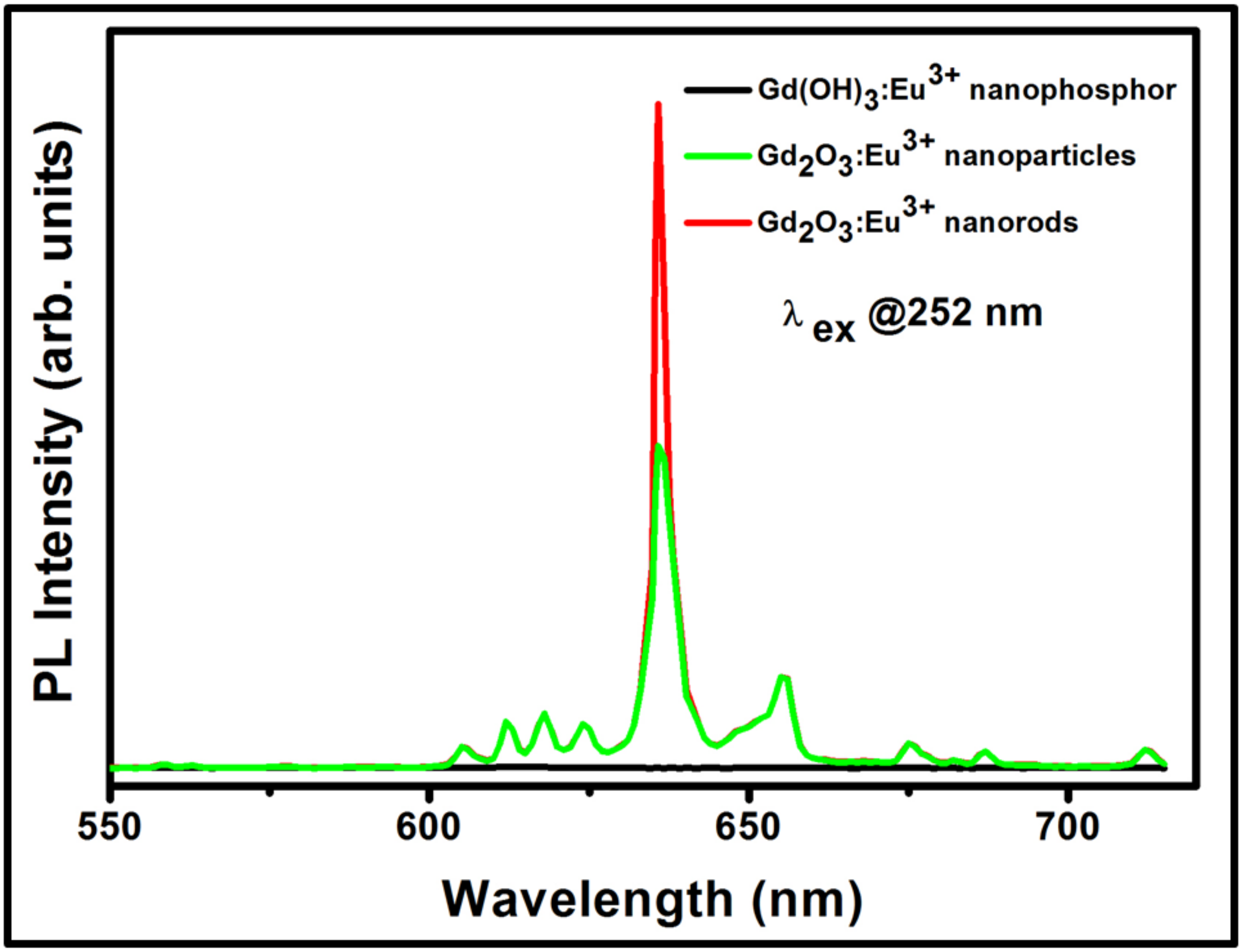

Figure S14.Combined effect of PL emission spectra of as-synthesized $Gd_2O_3:Eu^{3+}$ nanoparticles, $Gd(OH)_3:Eu^{3+}$ nanophosphor and $Gd_2O_3:Eu^{3+}$ nanorods under the excitation wavelength 252 nm.

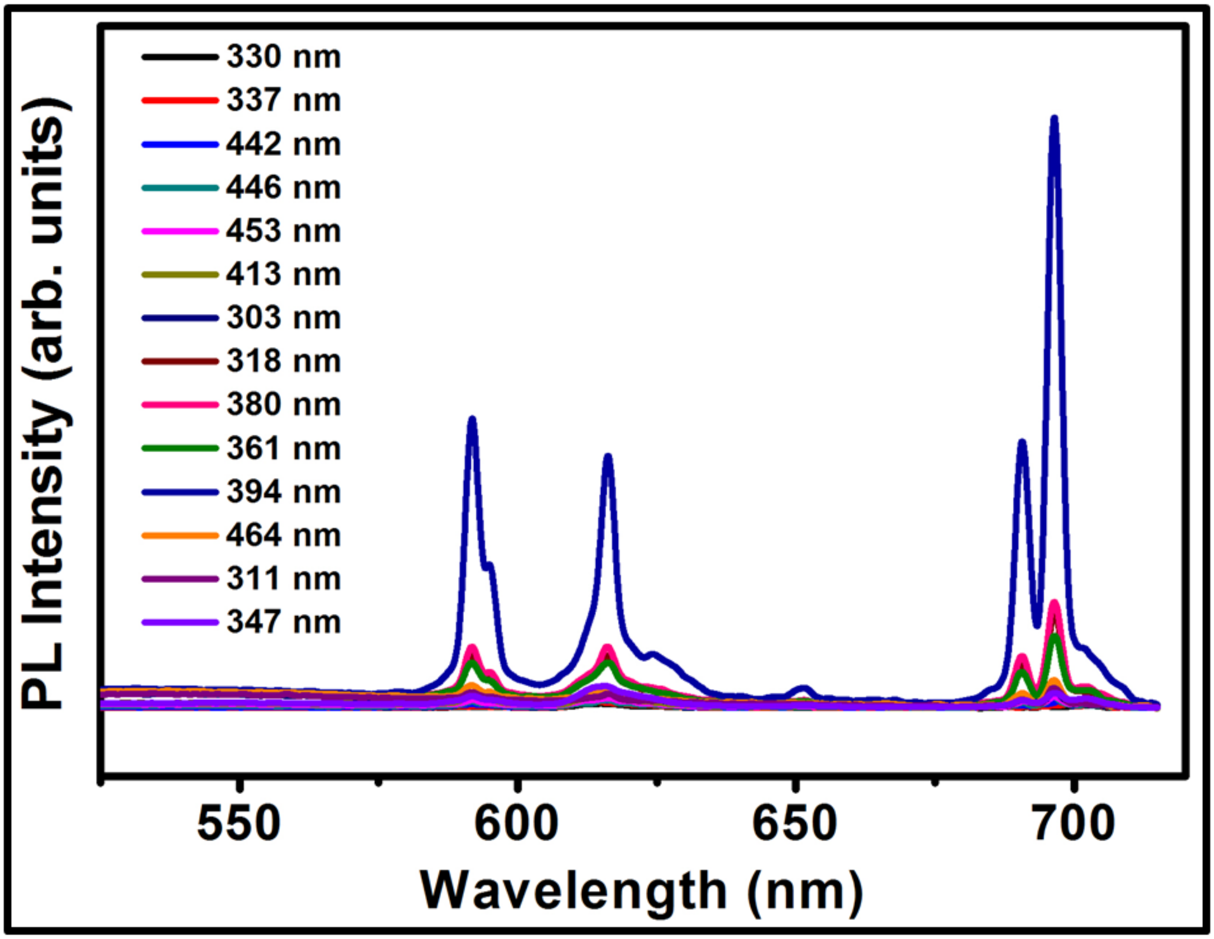

Figure S15. PL emission spectra of $Gd_{0.925}Eu_{0.075}(OH)_3$ nanophosphor under different excitation wavelengths.

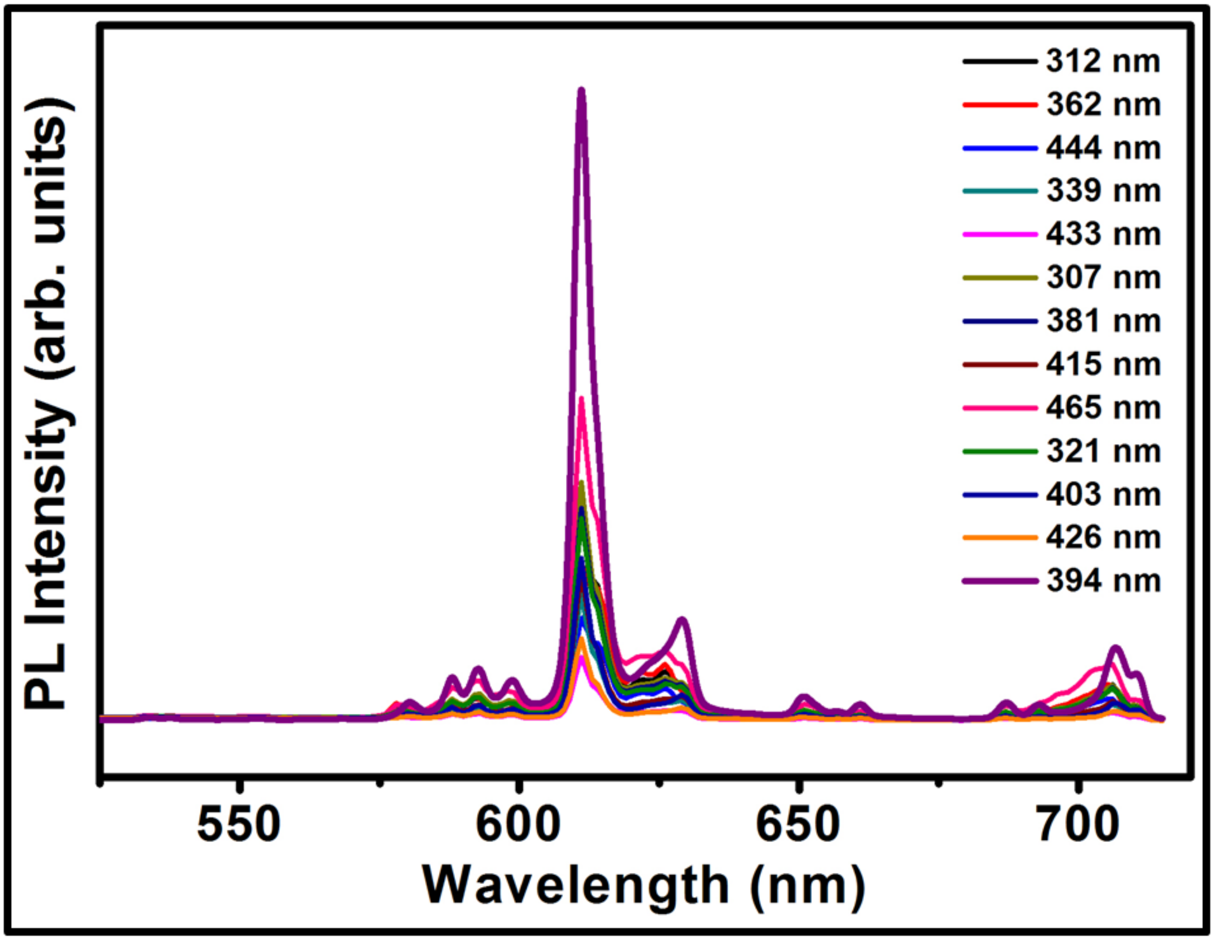

Figure S16. PL emission spectra of $Gd_{1.85}Eu_{0.15}O_3$ nanorods under different excitation wavelengths.

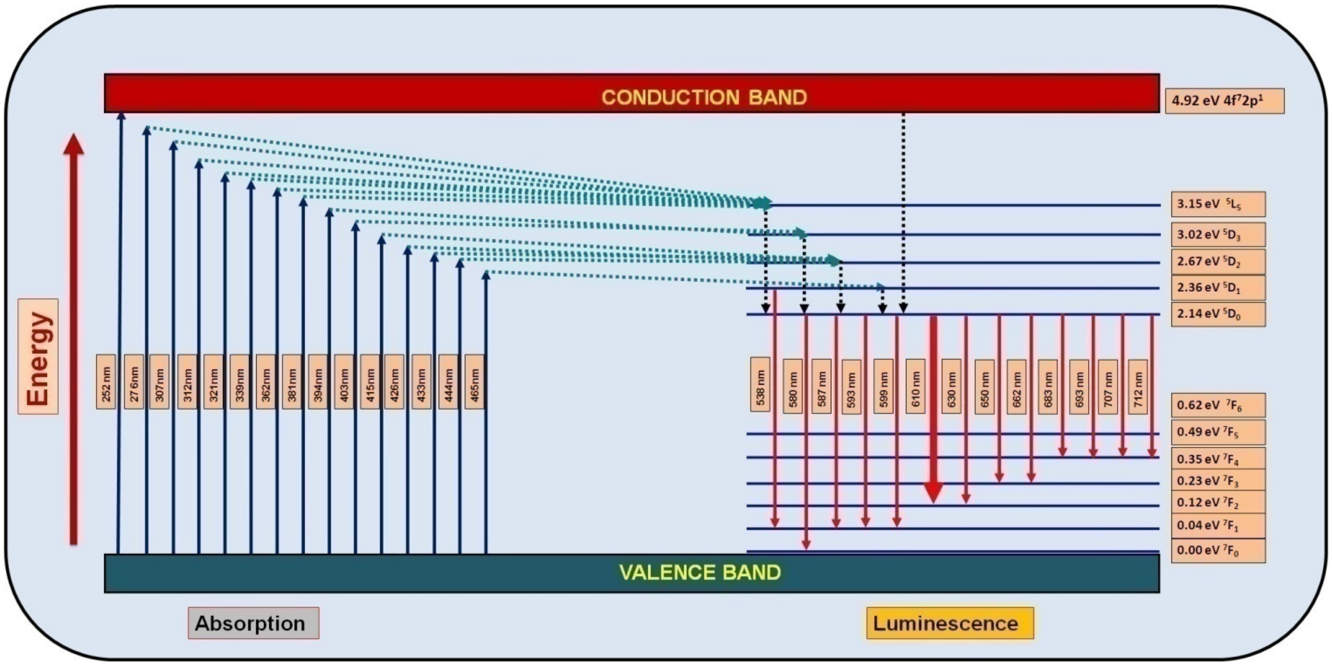

Figure S17. Energy level diagram with all possible transitions of Eu^{3+} ions in Gd_2O_3 system.

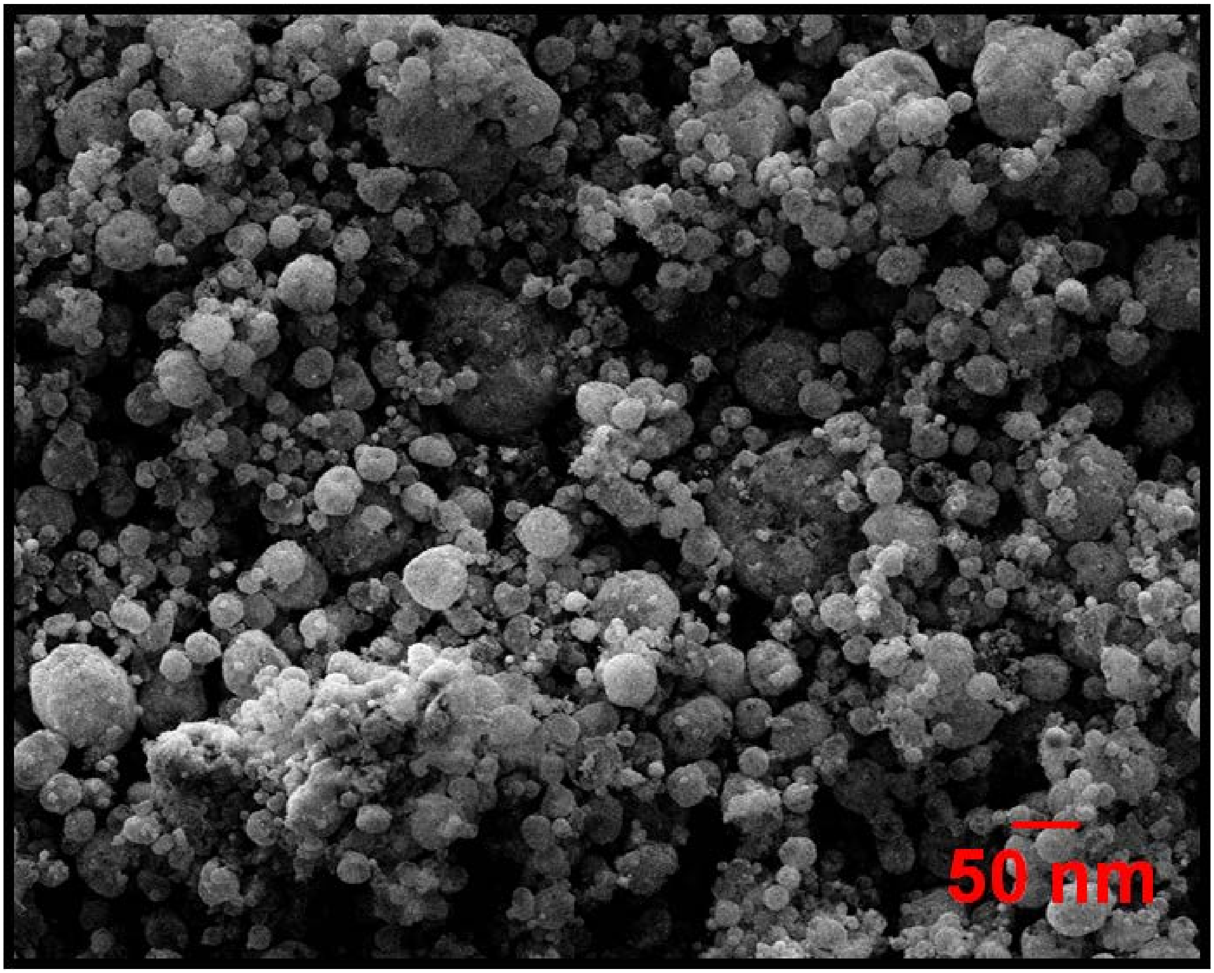

Figure S18. SEM image of as-synthesized $Gd_{1.85}Eu_{0.15}O_3$ spherical nanoparticles.

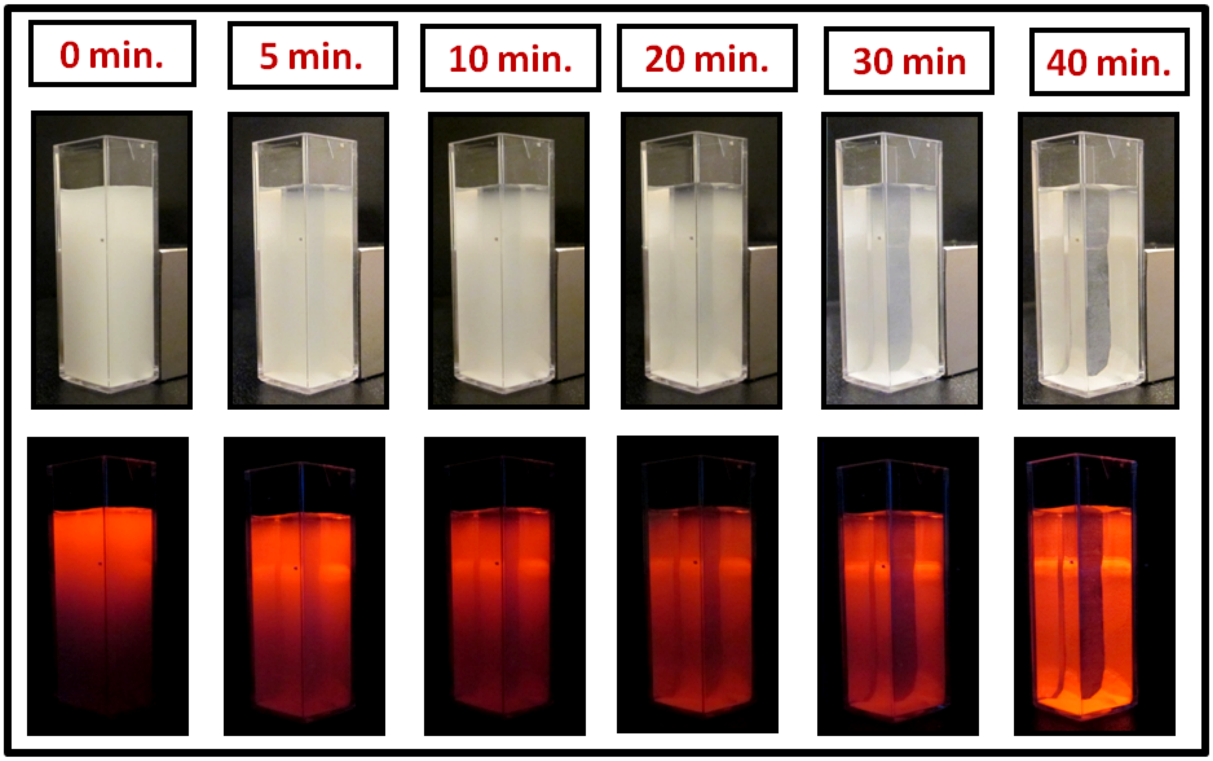

Figure S19. The slant view photographs of the magnetic tracking process of luminomagnetic $Gd_{1.85}Eu_{0.15}O_3$ nanorods in aqueous dispersion. It can be easily seen the deposition of nanorods on the cuvette wall after 40 minutes, where the magnet (~ 4000 Oe) was placed, both under room light and UV light. More specifically, photographs taken under UV light exhibit dark red color on the right side of the wall where the magnet is, after 40 min.

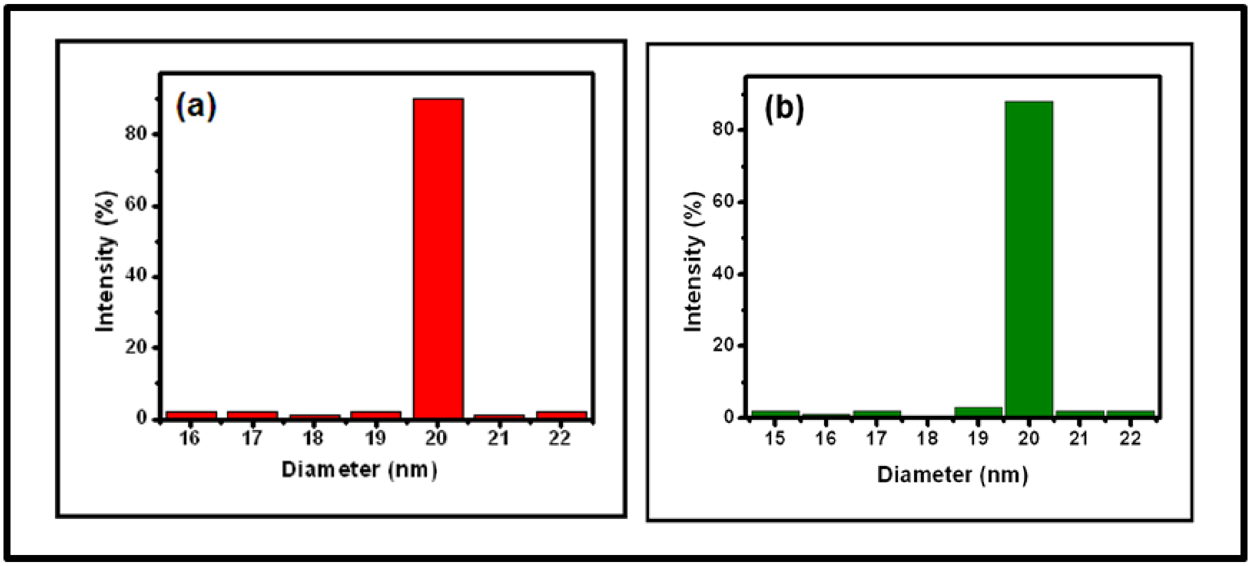

Figure S20. Diameter distributions plot obtained from DLS studies for $Gd_{1.85}Eu_{0.15}O_3$ nanorods dispersed in (a)Ethanol and (b) DI water.

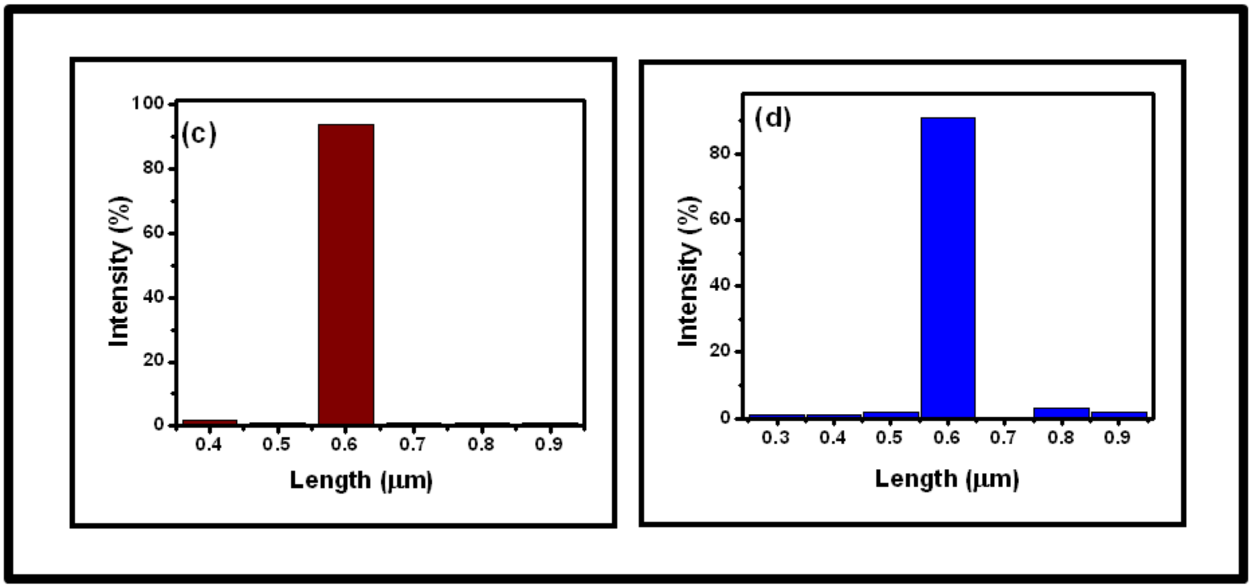

Figure S20. Length distributions plot obtained from DLS studies for $Gd_{1.85}Eu_{0.15}O_3$ nanorods dispersed in (c)Ethanol and (d) DI water.

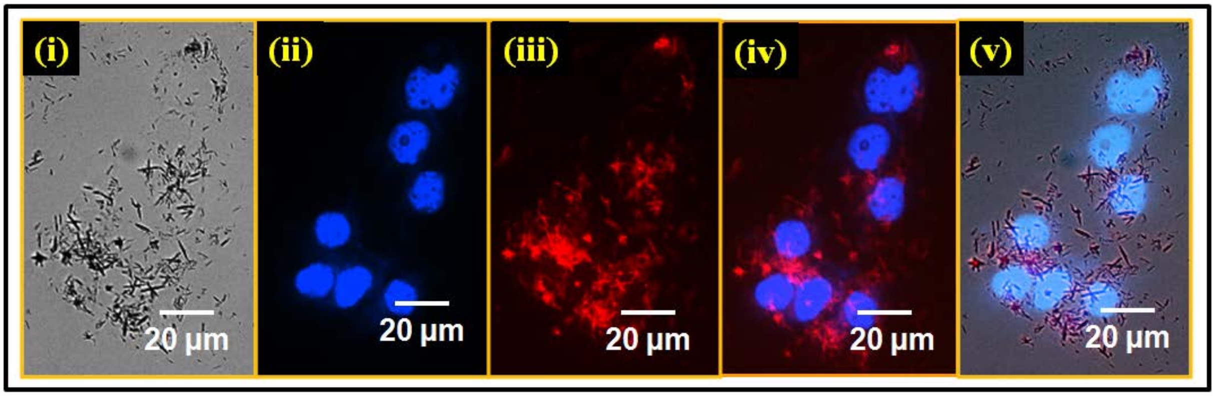

Figure S21. *In vitro* fluorescent microscopy images of MDA-MB-231 cells, cells incubated with $Gd_{1.85}Eu_{0.15}O_3$ nanorods (5μg mL^{-1}) for 4 hours. Sequential images show; (i). Phase contrast picture of MDA-MB-231 cells; (ii). Individual nucleus stained blue color with DAPI ; (iii). Red flurescence staining $Gd_{1.85}Eu_{0.15}O_3$ nanorods ;(iv). Overlapped images from blue DAPI and red $Gd_{1.85}Eu_{0.15}O_3$ nanorods images and (v) Overlap of phase contrast, blue and red from (i), (ii), and (iii) images respectively.

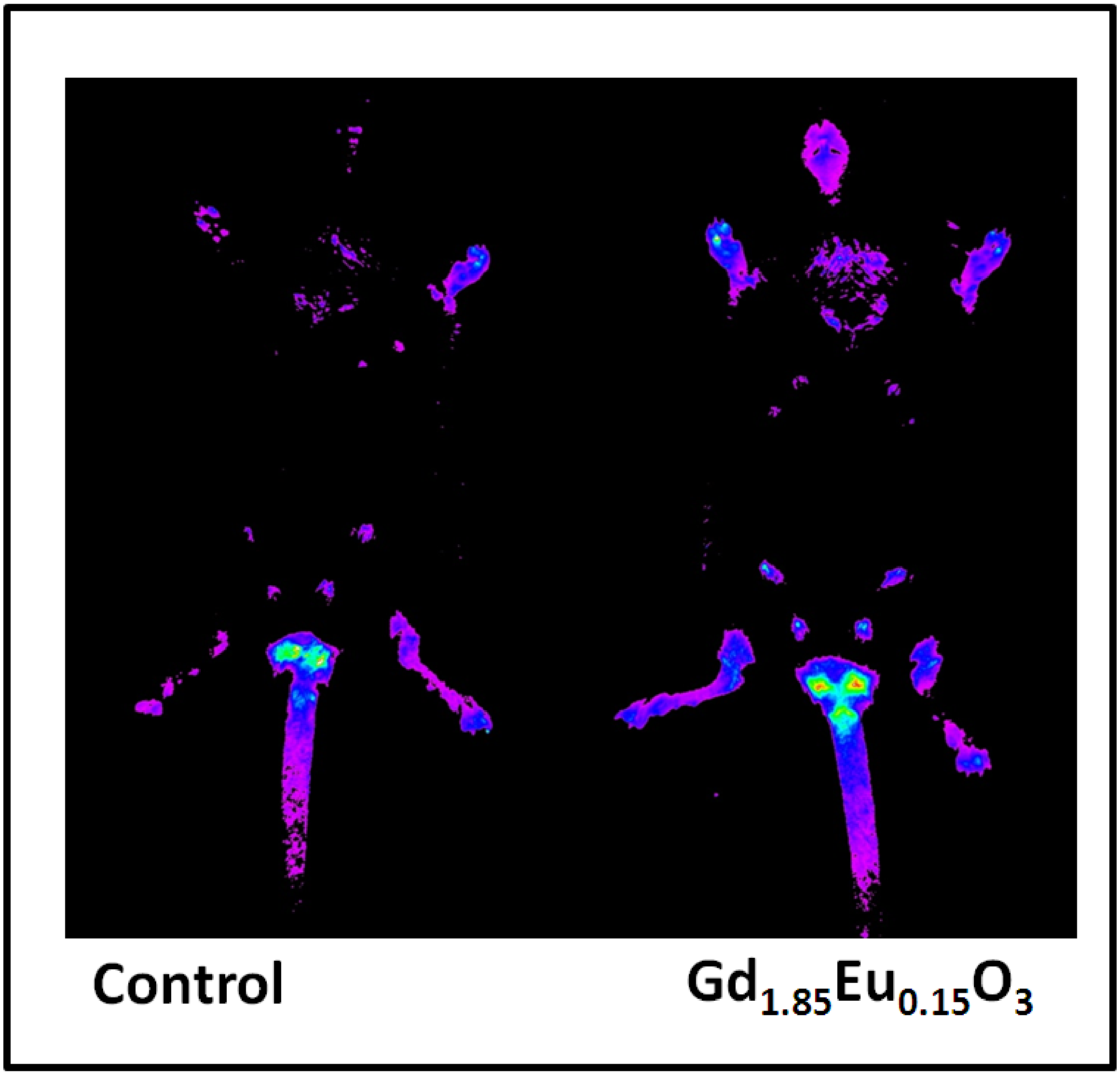

Figure S22. *In vivo* imaging in mice using $Gd_{1.85}Eu_{0.15}O_3$ nanorods. Six weeks old, C57BL/6J mice were anesthetized and injected with vehicle and $Gd_{1.85}Eu_{0.15}O_3$ nanorods intraperitoneally. Fluorescent images of whole body along with their controls were acquired by exciting at 495nm and emission was monitored at 600nm. Maximum intensity was observed at 60 min.

Table TS1. Least squares refined unit cell parameters and cell volume for variants of $Gd_{1-y}(OH)_3Eu_y^{3+}$ nanorods in the range y = 0.025 to 0.25.

$Gd_{1-y}Eu_y(OH)_3$ (y = 0.025 to 0.25)	Lattice parameter a=b(Å)	Lattice parameter c (Å)	Cell Volume (Å)^3
JCPDS Card No. 83-2037	6.3290	3.6310	125.9583
$Gd_{0.975}(OH)_3Eu_{0.025}^{3+}$	6.3300 ±0.0023	3.6339 ±0.0045	126.0951±0.2478
$Gd_{0.950}(OH)_3Eu_{0.050}^{3+}$	6.3318 ±0.0034	3.6353 ±0.0052	126.2191±0.3161
$Gd_{0.925}(OH)_3Eu_{0.075}^{3+}$	6.3365 ±0.0067	3.6388 ±0.0037	126.5283±0.3962
$Gd_{0.900}(OH)_3Eu_{0.100}^{3+}$	6.3352 ±0.0013	3.6378 ±0.0041	126.4416±.1944
$Gd_{0.875}(OH)_3Eu_{0.125}^{3+}$	6.3344 ±0.0038	3.6372 ±0.0019	126.3888±0.2177
$Gd_{0.850}(OH)_3Eu_{0.150}^{3+}$	6.3326 ±0.0072	3.6356 ±0.0057	126.2614±0.4851
$Gd_{0.825}(OH)_3Eu_{0.175}^{3+}$	6.3313±0.0046	3.6333 ±0.0032	126.1297±0.2944
$Gd_{0.800}(OH)_3Eu_{0.200}^{3+}$	6.3309 ±0.0033	3.6321 ±0.0026	126.0722±0.2217
$Gd_{0.775}(OH)_3Eu_{0.225}^{3+}$	6.3301 ±0.0027	3.6312 ±0.0049	126.0091±0.2775
$Gd_{0.750}(OH)_3Eu_{0.250}^{3+}$	6.3296 ±0.0048	3.6303 ±0.0091	125.9579±0.5068

Table TS2. Least squares refined unit cell parameters and cell volume for variants of $Gd_{2-x}O_3Eu^{3+}$ nanorods in the range x = 0.05 to 0.5.

$Gd_{2-x}Eu_xO_3$ (x = 0.05 to 0.5)	Lattice parameter a=b=c (Å)	Cell Volume (Å)^3
JCPDS Card No. 86-2477	10.80	1259.7120
$Gd_{1.95}O_3Eu_{0.05}^{3+}$	10.7711±0.0011	1249.6263±0.3828
$Gd_{1.90}O_3Eu_{0.10}^{3+}$	10.7934±0.0035	1257.4039±1.2232
$Gd_{1.85}O_3Eu_{0.15}^{3+}$	10.8101±0.0029	1263.2495±.1.0167
$Gd_{1.80}O_3Eu_{0.20}^{3+}$	10.7887±0.0046	1255.7620±1.6063
$Gd_{1.75}O_3Eu_{0.25}^{3+}$	10.7703±0.0082	1249.3479±2.8536
$Gd_{1.70}O_3Eu_{0.30}^{3+}$	10.7688±0.0028	1248.8260±0.9741
$Gd_{1.65}O_3Eu_{0.35}^{3+}$	10.7545±0.0035	1243.8576±1.2144
$Gd_{1.60}O_3Eu_{0.40}^{3+}$	10.7499±0.0029	1242.2622±1.0054
$Gd_{1.55}O_3Eu_{0.45}^{3+}$	10.7412±0.0045	1239.2485±1.5575
$Gd_{1.50}O_3Eu_{0.50}^{3+}$	10.7386±0.0098	1238.3488±3.3903

Table TS3. Intensity ratio of Gd^{3+}/Eu^{3+} estimated from different XPS survey scan spectra of $Gd_{2-x}Eu_xO_3$ (x = 0.05 to 0.5) .

**$Gd_{2-x}Eu_xO_3$ (x = 0.05 to 0.5)**	**Intensity Ratio Gd^{3+}/Eu^{3+}**
$Gd_{1.95}O_3Eu_{0.05}^{3+}$	~38.1
$Gd_{1.90}O_3Eu_{0.10}^{3+}$	~18.6
$Gd_{1.85}O_3Eu_{0.15}^{3+}$	~11.6
$Gd_{1.80}O_3Eu_{0.20}^{3+}$	~8.8
$Gd_{1.75}O_3Eu_{0.25}^{3+}$	~6.7
$Gd_{1.70}O_3Eu_{0.30}^{3+}$	~6.2
$Gd_{1.65}O_3Eu_{0.35}^{3+}$	~4.5
$Gd_{1.60}O_3Eu_{0.40}^{3+}$	~3.9
$Gd_{1.55}O_3Eu_{0.45}^{3+}$	~3.2
$Gd_{1.50}O_3Eu_{0.50}^{3+}$	~2.9

Table TS4. The comparative results of magnetic tracking application for Spherical shaped $Gd_{1.85}O_3Eu_{0.15}^{3+}$ nanoparticles, Flower-shaped $Gd_{0.925}(OH)_3Eu_{0.075}^{3+}$ hydroxide nanophosphor and $Gd_{1.85}O_3Eu_{0.15}^{3+}$ rod- shaped oxide nanophosphors.

**Morphology of nanophosphor**	**Tracking time**
Spherical shaped $Gd_{1.85}O_3Eu_{0.15}^{3+}$ nanoparticles	120 minutes
Flower-shaped $Gd_{0.925}$ $(OH)_3Eu_{0.075}^{3+}$ hydroxide nanophosphor	80 minutes
Rod –shaped $Gd_{1.85}O_3Eu_{0.15}^{3+}$ oxide nanophosphor	40 minutes
